# Immunotyping the Tumor Microenvironment Reveals Molecular Heterogeneity for Personalized Immunotherapy in Cancer

**DOI:** 10.1002/advs.202417593

**Published:** 2025-05-28

**Authors:** Dongqiang Zeng, Yunfang Yu, Wenjun Qiu, Qiyun Ou, Qianqian Mao, Luyang Jiang, Jianhua Wu, Jiani Wu, Huiyan Luo, Peng Luo, Wenchao Gu, Na Huang, Siting Zheng, Shaowei Li, Yonghong Lai, Xiatong Huang, Yiran Fang, Qiongzhi Zhao, Rui Zhou, Huiying Sun, Wei Zhang, Jianping Bin, Yulin Liao, Masami Yamamoto, Tetsuya Tsukamoto, Sachiyo Nomura, Min Shi, Wangjun Liao

**Affiliations:** ^1^ Cancer Center The Sixth Affiliated Hospital School of Medicine South China University of Technology Foshan 528000 China; ^2^ Foshan Key Laboratory of Translational Medicine in Oncology The Sixth Affiliated Hospital School of Medicine South China University of Technology Foshan 528000 China; ^3^ Department of Oncology Nanfang Hospital Southern Medical University Guangzhou Guangdong 510515 P. R. China; ^4^ Guangdong Provincial Key Laboratory of Malignant Tumor Epigenetics and Gene Regulation Guangdong‐Hong Kong Joint Laboratory for RNA Medicine Department of Medical Oncology Sun Yat‐sen Memorial Hospital Sun Yat‐sen University Guangzhou 510120 China; ^5^ Guangdong Provincial Key Laboratory of Cancer Pathogenesis and Precision Diagnosis and Treatment, Joint Big Data Laboratory, Department of Medical Oncology, Shenshan Medical Center Sun Yat‐sen Memorial Hospital Sun Yat‐sen University Shanwei 516600 China; ^6^ Faculty of Medicine Macau University of Science and Technology Taipa Macao 999078 China; ^7^ Department of Breast Surgery The First Affiliated Hospital Jinan University Guangzhou 510630 China; ^8^ Sun Yat‐sen University Cancer Center State Key Laboratory of Oncology in South China Collaborative Innovation Center for Cancer Medicine Guangzhou Guangdong 510060 P. R. China; ^9^ Department of Oncology Zhujiang Hospital Southern Medical University Guangzhou Guangdong 510280 China; ^10^ Department of Artificial Intelligence Medicine Graduate School of Medicine Chiba University Chiba 260‐8677 Japan; ^11^ Department of Cardiology Nanfang Hospital Southern Medical University Guangzhou Guangdong 510515 P. R. China; ^12^ Laboratory of Physiological Pathology School of Veterinary Nursing and Technology Nippon Veterinary and Life Science University Tokyo 180‐8602 Japan; ^13^ Department of Diagnostic Pathology Fujita Health University School of Medicine Toyoake Aichi 470‐1192 Japan; ^14^ Department of Gastrointestinal Surgery Graduate School of Medicine The University of Tokyo Tokyo 113‐8655 Japan

**Keywords:** cancer, immunotherapy, immunotyping, IL‐1, tumor microenvironment

## Abstract

The tumor microenvironment (TME) significantly influences cancer prognosis and therapeutic outcomes, yet its composition remains highly heterogeneous, and currently, no highly accessible, high‐throughput method exists to define it. To address this complexity, the TMEclassifier, a machine‐learning tool that classifies cancers into three distinct subtypes: immune Exclusive (IE), immune Suppressive (IS), and immune Activated (IA), is developed. Bulk RNA sequencing categorizes patient samples by TME subtype, and in vivo mouse model validates TME subtype differences and differential responses to immunotherapy. The IE subtype is marked by high stromal cell abundance, associated with aggressive cancer phenotypes. The IS subtype features myeloid‐derived suppressor cell infiltration, intensifying immunosuppression. In contrast, the IA subtype, often linked to EBV/MSI, exhibits robust T‐cell presence and improved immunotherapy response. Single‐cell RNA sequencing is applied to explore TME cellular heterogeneity, and in vivo experiments demonstrate that targeting IL‐1 counteracts immunosuppression of IS subtype and markedly improves its responsiveness to immunotherapy. TMEclassifier predictions are validated in this prospective gastric cancer cohort (TIMES‐001) and other diverse cohorts. This classifier could effectively stratify patients, guiding personalized immunotherapeutic strategies to enhance precision and overcome resistance.

## Graphical abstract

This study develops TMEclassifier, a machine‐learning tool that classifies cancers into three distinct subtypes—Immune exclusive (IE), immune suppressive (IS), and immune activated (IA)—which exhibit significant heterogeneity and necessitate customized therapeutic strategies. This TME immunotyping system holds promise for advancing personalized precision medicine for cancer patients.

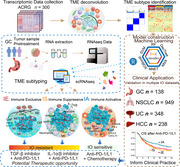

## 1 Introduction

Targeting PD‐1/L1 with immune checkpoint blockade (ICB) has demonstrated promising efficacy in antitumor treatment and has been approved as a clinical option. The Phase III CheckMate‐649 trial recently achieved its primary objective and reported encouraging findings. First‐line therapy with nivolumab plus chemotherapy yielded significant improvements in overall survival (OS) and progression‐free survival (PFS) compared to chemotherapy alone. However, the results also indicated that only a subset of patients exhibited a positive response to nivolumab plus chemotherapy, suggesting that many patients may not benefit from this treatment.^[^ ^1^ ^]^ Likewise, numerous studies have identified a high proportion of patients who are intrinsically resistant to ICBs.^[^ ^2^ ^]^ Hence, it is imperative to identify non‐responsive patients and investigate mechanisms of drug resistance in cancer patients.

Classification of cancers is helpful to explain the biological characteristics heterogeneity of different patients and guide personalized therapeutic regimens. A notable example is the consensus molecular subtypes classifier, which has demonstrated significant utility in scientific research and clinical settings for colorectal cancer.^[^ ^3^ ^]^ Another successful classifier, the PAM50 classifier, effectively stratifies breast cancers and has dramatically influenced clinical treatment decisions. Additionally, the STIM classifier for intrahepatic cholangiocarcinoma categorizes tumors into five subtypes, each associated with distinct potential treatment regimens.^[^ ^4^ ^]^ Gastric cancer exhibits significant heterogeneity, primarily manifested in the diverse infiltration proportions and biological functions of tumor cells, immune cells, stromal cells, and other essential components. The current well‐known gastric cancer classification systems, such as The Cancer Genome Atlas^[^ ^5^ ^]^ (TCGA) and the Asian Cancer Research Group^[^ ^6^ ^]^ (ACRG) molecular subtypes, have provided valuable insights.

However, these classifications do not fully consider the various cell types within the tumor microenvironment (TME), which remains a limitation to some extent. Numerous studies have emphasized the correlation between the TME and treatment response.^[^ ^7^ ^]^ Understanding the heterogeneity of TME can help optimize treatment strategies and improve patient outcomes. In previous research, we developed a robust TME evaluation system^[^ ^8^ ^]^ (TMEscore), demonstrating its effectiveness in predicting immunotherapeutic outcomes.^[^ ^9^ ^]^ Therefore, a comprehensive evaluation of the TME is crucial for predicting the efficacy of immunotherapy. Nevertheless, there is a scarcity of studies focusing on gastric cancer classification based on the infiltrating patterns of the TME, and a reliable TME‐based classification system has yet to be established.

In this study, we have successfully identified three conserved subtypes of TME in cancer. Subsequently, we developed an open‐access R package called *TMEclassifier* (https://github.com/LiaoWJLab/TMEclassifier), which utilizes transcriptomic data to classify the TME subtypes. These subtypes exhibited distinct infiltration patterns, biological characteristics, clinical prognosis, and response to immunotherapy. Notably, the immune‐activated (IA) subtype demonstrated a more favorable prognosis and higher efficacy in immunotherapy. These findings were further validated in diverse cancer immunotherapy cohorts. The comprehensive analysis results strongly support the effectiveness of TMEclassifier in accurately classifying cancer into three distinct TME subtypes, identifying patients likely to respond to immunotherapy, and facilitating the identification of potential targetable vulnerabilities. These advancements will significantly contribute to the study of immunotherapy and enable personalized and precise treatment approaches.

## 2 Results

### 2.1 Development and Evaluation of a Robust TME Subtyping Model for Cancer

**Figure 1:**
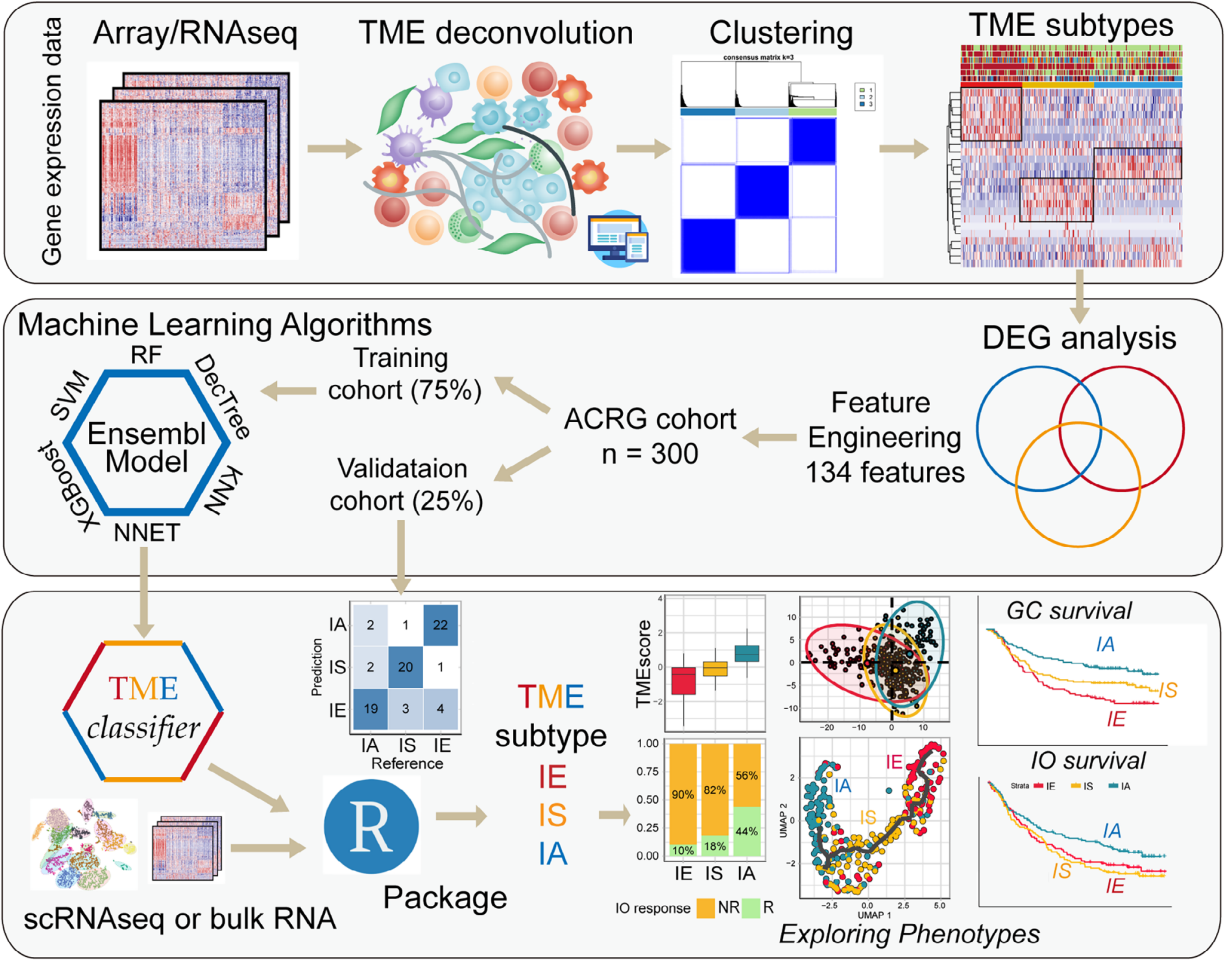
Workflow and Graphical methodological overview of the TMEclassifier construction and TME subtype‐specific analysis. ACRG, The Asian Cancer Research Group; TME, tumor microenvironment; IE, Immune Exclusive (red), IS, Immune Suppressive (orange), IA, Immune Activated (blue); TME, Tumor Microenvironment; SVM, support vector machine; RF, random forest; NNET, neural network; XGBoost, extreme gradient boost; DecTree, decision tree; KNN, K nearest neighbor; scRNAseq, single‐cell RNA sequencing; GC, gastric cancer. IO, Immunotherapy.

By inputting the gene expression matrix from the ACRG gastric cancer cohort,^[^ ^6^ ^]^ we estimated the infiltration of 23 TME cells using CIBERSORT and MCP‐counter.^[^ ^10^ ^]^ Following unsupervised clustering analysis, we identified three robust patterns of TME infiltration: immune exclusive (IE), immune suppressive (IS), and immune activated (IA).^[^ ^8^ ^]^ To streamline the procedures, enhance the reproducibility of TME subtyping, and investigate the characteristics of each subtype, we developed a convenient, efficient, and reliable TME subtype classification model (**Figure** **1**). Initially, we conducted pairwise differential expression gene (DEG) analysis to identify DEGs specific to each subtype in the ACRG cohort. After performing feature engineering, we selected 134 feature genes for TME subtype classification (IE: 40, IS: 19, and IA: 75; Table S1, Supporting Information). The expression of each gene group was notably elevated in the corresponding subtype (Figure S1A and Table S1, Supporting Information). Subsequently, we divided the tumors in the ACRG cohort into a training set (75%) and a validation set (25%), and employed six machine learning algorithms (SVM, RF, NNET, XGBoost, DecTree, and KNN) to jointly train the model using the training set. Finally, we integrated the six models into an ensemble model for TME subtype classification, achieving high concordance in the training cohort (accuracy: 94%, Kappa value: 90%, Figure S1B, Supporting Information) and internally validated cohort (accuracy: 82%, Kappa value: 74%, Figure S1C, Supporting Information). We then applied the TMEclassifier to multiple cancer cohorts, including 1992 gastric cancers analyzed in this study (classification results shown in Table S2, Supporting Information). We compared the three TME subtypes to identify distinctive characteristics at the TME, signaling pathway, cell‐cell interaction, and metabolism levels using bulk‐seq and single‐cell RNA‐seq data. Additionally, we aimed to explore the clinical translational value of this classification system.

### 2.2 Association of TME Subtypes with Clinical Features, Survival Outcomes, Molecular Characteristics, and Immunotherapy Efficacy in Gastric Cancer

**Figure 2:**
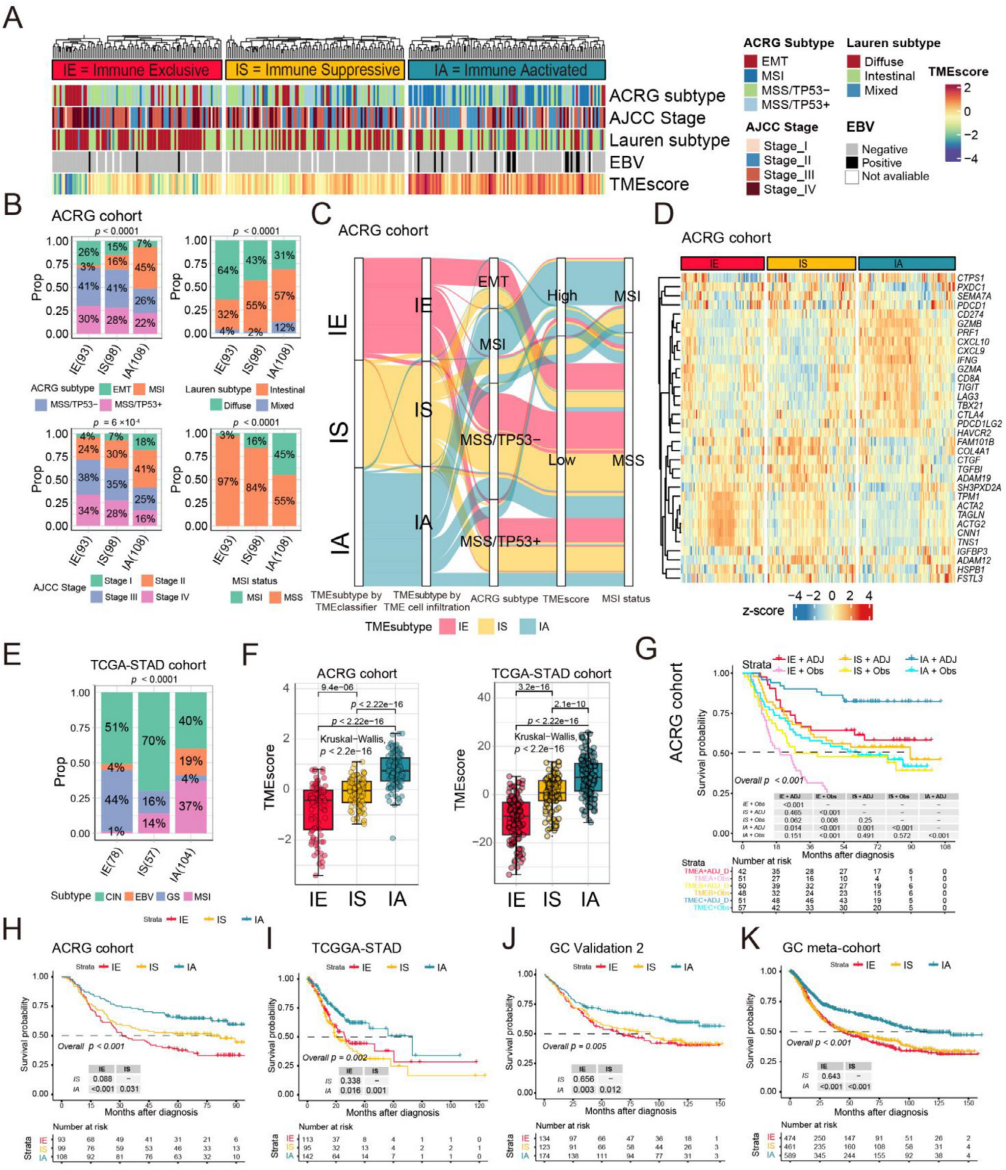
Association of TME subtypes with clinical features, immunotherapy efficacy biomarkers, and survival outcomes. A) Heatmap showing clinical information, including ACRG subtypes, AJCC stage, Lauren subtypes, *EBV* status and TMEscore across three TME subtypes identified by the TMEclassifier. B) Proportion of different clinical characteristics among the three TME subtypes in ACRG cohort shown by bar plots. C) Alluvial diagram depicting the relationship between TME subtypes by TMEclassifier, TME subtypes by TME cell infiltration, ACRG subtypes, TMEscore and MSI status. D) Heatmap of the expression of immune‐ and stromal‐related genes among three TME subtypes in ACRG cohort. E) The TCGA subtypes proportion among three TME subtypes in TCGA‐STAD cohort shown by bar plot. F) Box plots of difference in TMEscore among TME subtypes in ACRG cohort and TCGA‐STAD cohort. G) Kaplan–Meier curves of overall survival (OS) for patients in subgroups stratified by TME subtypes and receipt of adjuvant therapy of patients from ACRG cohort. H–K) Survival analysis of patients by TME subtypes in the gastric cancer cohorts including the ACGR cohort (H), TCGA‐STAD cohort (I), GSE84437 cohort (J), and GC meta‐cohort (K). In box plots, *p* values were calculated using the Mann–Whitney test for comparisons between two groups, and the Kruskal‐Wallis test was used to calculate *p* values for comparisons of more than two groups. In bar plots, the Chi‐squared test *p* values were displayed at the top of the figure. The log‐rank test was used to assess the statistical significance of the prognostic differences among the subtypes in the survival analyses above. EMT, epithelial–mesenchymal transition; MSI, microsatellite instability; MSS, microsatellite stable; AJCC, The American Joint Committee on Cancer; EBV, Epstein‐Barr virus; TCGA, The Cancer Genome Atlas; STAD, stomach adenocarcinoma; CIN, chromosomal instability; GS, genomically stable; ADJ, adjuvant therapy; Obs, observation.

Upon utilizing the TME subtypes model, we stratified tumors from the ACRG cohort into three distinct groups: IE (*n* = 93), IS (*n* = 98), and IA (*n* = 108). When comparing the clinical characteristics among these TME subtypes, we made noteworthy observations. The IE subtype, which exhibited the worst prognosis, was predominantly enriched in the epithelial‐mesenchymal transition (EMT) subtype, whereas the IA subtype, associated with a better prognosis, displayed a higher prevalence of microsatellite instability (MSI) and Epstein–Barr virus (EBV) infectious tumors (*p* < 0.0001). The IE subtype had the highest proportion of the diffuse Lauren subtype (64%, *p* < 0.0001), while the IA subtype exhibited the lowest proportion (31%, *p* < 0.0001). Furthermore, the IA subtype was predominantly characterized by EBV infection. Notably, a significant proportion of IE patients were classified as AJCC stage III and IV (III: 38%, IV: 34%), whereas a higher percentage of early‐stage patients (I: 18%, II: 41%, *p =* 6 ×10^−4^) were observed in the IA subtype (**Figure** **2**A,B). In the ACRG cohort, the TME subtypes identified by the TMEclassifier, utilizing the expression of feature genes, showed a high concordance with the TME clusters classified based on the 23 TME cells (Figure 2C; Figure S1B,C, Supporting Information). This suggests that TMEclassifier can efficiently and accurately identify TME subtypes. Additionally, nearly half of the IA subtype belonged to the MSI subtype, and the majority of patients in the TMEscore‐high group were enriched in the IA subtype. The IE subtype mainly consisted of the EMT and MSS subtypes (*p* < 0.0001) and exhibited a low TMEscore (Figure 2B,C).

We examined immune‐relevant gene signatures obtained from the IMvigor210 study^[^ ^11^ ^]^ and observed that the IA subtype exhibited high expression levels of multiple immune checkpoint genes (*CD274*, *PDCD1LG2*, *TIGIT*, *LAG3*, *CTLA4*, and *HAVCR2*), as well as genes associated with effector CD8^+^ T cell function (*CD8A*, *GZMA*, *GZMB*, *CXCL9*, *CXCL10*, *IFNG*, *PRF1*, and *TBX21*; Figure 2D). These findings suggest that IA subtype may possess enhanced T cell‐mediated antitumor activity and increased sensitivity to ICB therapies. In the TCGA‐STAD cohort, over half of the IA subtype tumors were classified as microsatellite instability (MSI) and Epstein‐Barr virus (EBV) subtypes (37% + 19%), both of which are associated with improved prognosis and response to ICB therapies^[^ ^12^ ^]^ (*p* < 0.0001; Figure 2E). Furthermore, IA subtype demonstrated a higher tumor mutation burden (TMB) in both the ACRG and TCGA‐STAD cohorts, indicating that IA tumors might exhibit a higher number of neoantigens, leading to increased immune activation and susceptibility to ICB therapies^[^ ^13^ ^]^ (*p* = 1.9×10^−8^ and *p* < 2.2×10^−16^, respectively; Figure S2A, Supporting Information). Additionally, there was an increasing trend in the TMEscore from IE to IS to IA, with IA showing the highest TMEscore (both *p* < 2.2×10^−16^, Figure 2F; *p* < 2.2×10^−16^, *p* < 2.2×10^−16^, and *p* = 3.6×10^−10^, respectively; Figure S2B, Supporting Information). Our previous studies have demonstrated that a higher TMEscore is associated with more favorable progression‐free survival following anti‐PD‐1 immunotherapy combined with chemotherapy, strongly suggesting that TME subtypes can serve as predictive markers for immunotherapy response in gastric cancer.

Survival analysis conducted on the ACRG cohort demonstrated that IA tumors exhibited the most favorable overall survival, while IE tumors had the poorest outcome, and IS tumors displayed an intermediate prognosis (Figure 2H). In the TCGA‐STAD, GSE84437, GSE34942, and meta‐cohort (which combined 1524 samples from multiple gastric cancer cohorts), IA tumors still exhibited significantly better prognosis compared to IE or IS tumors, even though there was no significant difference between IE and IS (*p* = 0.002, *p* = 0.005, and *p* < 0.001, respectively; Figure 2I–K; *p* = 0.006; Figure S2C, Supporting Information). However, in the other two cohorts analyzed for TME subtyping (GSE57303, *p* = 0.088; and GSE15459, *p* = 0.857, respectively; Figure S2D,E, Supporting Information), a significant difference was not observed. Interestingly, our observations revealed that adjuvant therapy (chemotherapy and/or radiotherapy) significantly improved the overall survival of patients with IE and IA tumors compared to the observation group (IE: *p* < 0.001, IA: *p* < 0.001). However, adjuvant therapy did not confer survival benefits to patients with the IS subtype in the ACRG cohort (*p* = 0.25; Figure 2G), highlighting the inherent resistance of IS tumors to adjuvant chemotherapy and radiotherapy.

In both the ACRG and TCGA‐STAD cohorts, IA gastric cancers exhibited the highest tumor mutation burden (TMB), indicating a potential abundance of somatic mutations and neo‐antigens in IA tumors. We compared the mutation frequencies of various genes among the three TME subtypes in both the ACRG and TCGA‐STAD cohorts to further investigate the molecular characteristics. The results, sorted by adjusted *p* value, demonstrated statistically significant differences (Figure S2F,G, Supporting Information). Previous studies have highlighted the significance of mutations in specific genes such as *ARID1A*,^[^ ^14^ ^]^ *PIK3CA*,^[^ ^15^ ^]^ *KMT2D*, and *SYNE1* in influencing the response to immunotherapy with ICBs.^[^ ^16^ ^]^ Our previous data also indicated that mutations in *ARID1A* and *PIK3CA* were associated with increased immune infiltration and higher TMEscore in gastric cancer,^[^ ^9^ ^]^ which could potentially enhance the efficacy of anti‐PD‐1/PD‐L1 immunotherapy. These findings align with previous research, further supporting that specific gene mutations may influence the response to immunotherapy. Collectively, our data suggest that TME subtyping is associated with clinical and pathological characteristics, molecular subtypes, and survival outcomes in gastric cancer. This classification approach holds the potential for optimizing individualized therapy for patients with gastric cancer.

### 2.3 Distinct Infiltration Patterns of TME Cells in Three Subtypes

**Figure 3:**
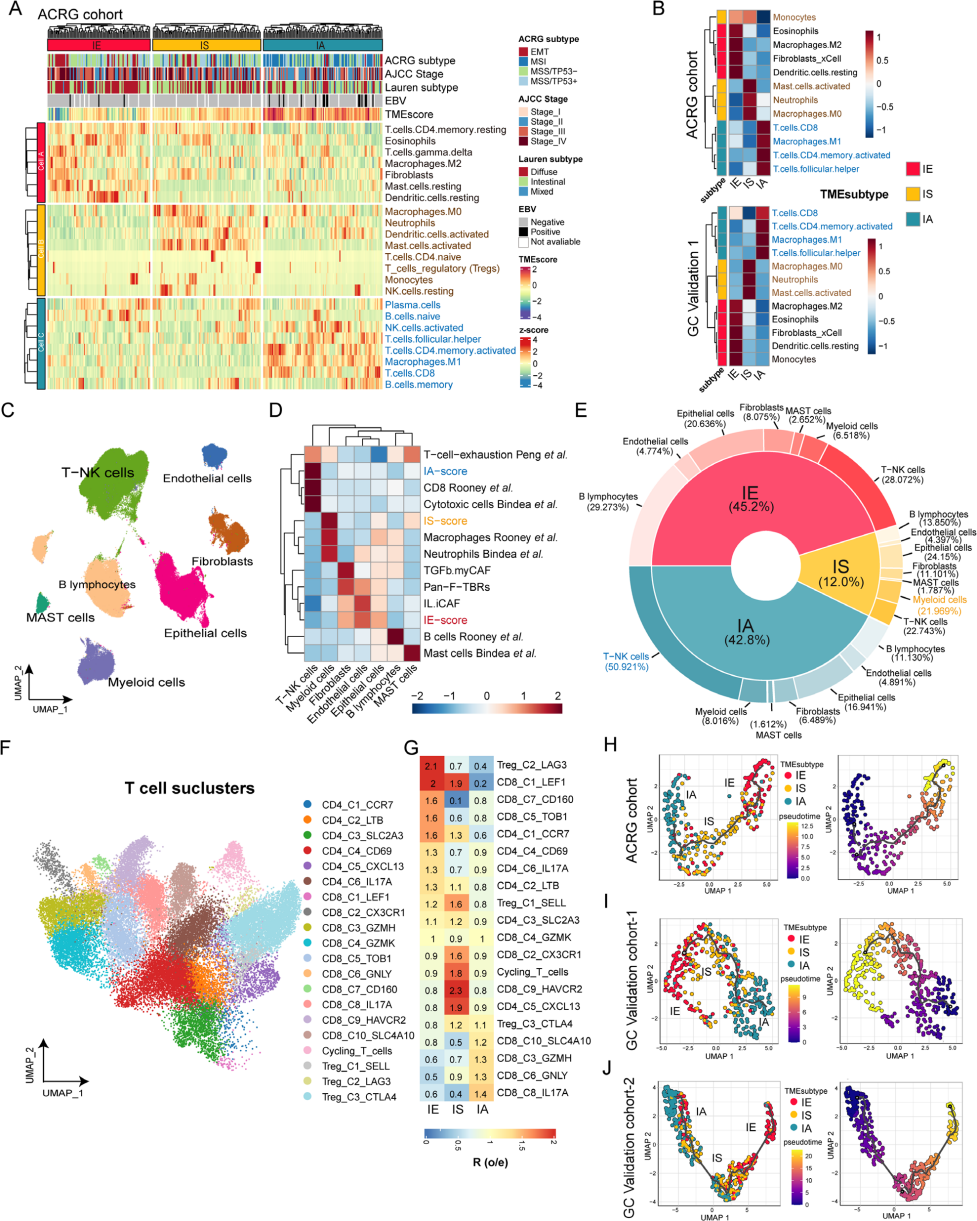
TME landscape and dynamic evolution of the three TME subtypes. A) Heatmap showing the distribution of 300 samples from the ACRG cohort across three TME subtypes identified by TME classifier (columns) and the infiltration levels of the 23 immune cell types in three TME subtypes (rows). B) Different infiltrated cell types among three TME subtypes in ACRG cohort (upper) and TCGA‐STAD cohort (GC Validation 1, bottom). C) Uniform manifold approximation and projection (UMAP) plot of the annotations for seven basal cell types from single cell RNA‐seq data of 40 gastric cancer samples. D) Heatmap comparing the expression of different cell types scores in the seven basal cell clusters. E) Donut plot showing the proportion of three TME subtypes (inner circle) and the comparison of seven basal cell clusters of each TME subtype (outer circle) from scRNA‐seq data. F) T cell landscape of TME subtypes at single‐cell resolution (OMIX001073, *n* = 10). UMAP plot annotated with T cell types from single‐cell RNA‐seq data of ten gastric cancer samples. G) Heatmap showing the preferential distribution of T cell subgroups across different TME subtypes. H–J) UMAP plot showing pseudo time ordering of TME subtypes in the ACRG cohort (H), TCGA‐STAD cohort (GC Validation cohort‐1, I), and GSE84437 cohort (GC Validation cohort‐2, J). Each point corresponds to a sample and is color‐coded by TME subtypes (left) and differentiation score (right).

Distinct infiltration patterns of TME cells were observed in three TME subtypes classified by TMEclassifier. **Figure** **3**A,B, as well as Figure S4A (Supporting Information), illustrate these patterns. IE subtype exhibited increased infiltration of cancer‐associated fibroblasts (CAFs), endothelial cells, M2‐macrophages (not observed in TCGA‐STAD cohort, *p* = 0.12), eosinophils (not observed in TCGA‐STAD cohort, *p* = 0.39), and resting dendritic cells. IS subtype showed the highest infiltration of neutrophils, activated mast cells, and M0‐macrophages, but the least infiltration of CD8^+^ T cells. IA subtype had higher infiltration of CD8^+^ T cells, M1‐macrophages, activated memory CD4^+^ T cells, and follicular helper T cells (all the results were statistically significant except the two cells mentioned above, the *p* values are shown in the results respectively; Figure S3B–D, Supporting Information). Notably, CD8^+^ T cells and M1‐macrophages play a role in promoting inflammation and antitumor immunity,^[^ ^11^, ^17^ ^]^ suggesting that IA tumors may respond favorably to ICB.

To further validate these findings in greater detail, a single‐cell RNA‐seq dataset containing 40 gastric cancer samples^[^ ^7b^ ^]^ was analyzed. A machine learning algorithm was employed for single‐cell annotation, resulting in seven main clusters (B lymphocytes, Endothelial cells, Epithelial cells, Fibroblasts, Mast cells, Myeloid cells, and T‐NK cells; Figure 3C). The top 100 differentially expressed genes upregulated in each TME subtype and other cell type signatures from our previous research were utilized to estimate signature scores at the single‐cell level. Strikingly, the heatmap aggregated cells of the same type revealed that T‐NK cells had higher IA scores, certain myeloid cells exhibited increased IS scores, and endothelial cells and fibroblasts were associated with higher IE scores (Figure 3D). Subsequently, the patients were classified into the three TME subtypes through pseudo‐bulk analysis, and their cell proportions were compared. IS had the highest proportion of myeloid cells (21.969%), while T‐NK cells accounted for 50.921% of cells in IA (Figure 3E). In our investigation of the second gastric cancer single‐cell dataset (OMIX001073, *n* = 10), we employed the TMEclassifier to assign tumor microenvironment (TME) subtypes, resulting in the identification of 4 IA subtype samples, 2 IS subtype samples, and 4 IE subtype samples. According to the original data's cell annotation, this dataset was categorized into ten major cell types, including B lymphocytes, plasma cells, endothelial cells, epithelial cells, endocrine cells, fibroblasts, smooth muscle cells, mast cells, myeloid cells, and T‐NK cells (Figure S4A, Supporting Information). When comparing the distribution of predominant cell types across different TME subtypes, we observed a higher prevalence of fibroblasts in IE subtype samples, increased myeloid cells in IS subtype samples, and an enrichment of T‐NK cells in IA subtype samples, consistent with bulk transcriptome results (Figure S4B, Supporting Information). Further analysis involved re‐clustering of T cells, myeloid cells, and stromal cells to explore subpopulations within these major cell groups. Re‐clustering of T cells revealed 10 clusters of CD8^+^ T cells, 6 clusters of conventional CD4^+^ T cells, 3 Treg clusters, and 1 cycling T cell cluster, representing T cells currently in the cell cycle (Figure 3F).

Literature sources indicated that the CD8_C1_LEF1 and CD4_C1_CCR7 clusters represented naive T cells; CD8_C2_CX3CR1 expressed genes associated with effector functionality akin to effector T cells; CD8_C3_GZMH represented activated T cells; CD8_C4_GZMK related to effector memory cells; CD8_C5_TOB1 and CD8_C6_GNLY denoted tissue‐resident memory CD8 T cells; CD4_C2_LTB, CD4_C3_SLC2A3, and CD4_C4_CD69 were related to memory T cells; CD8_C9_HAVCR2 and CD4_C5_CXCL13 highly expressed immune checkpoint genes such as CTLA4, PDCD1, and TIGIT, markers known for T cell exhaustion; CD8_C7_CD160 comprised intraepithelial lymphocytes; and CD8_C10_SLC4A10 was characterized as mucosal‐associated invariant T cells. The three Treg subtypes, Treg_C1_SELL, Treg_C2_LAG3, and Treg_C3_CTLA4, displayed characteristic co‐expression of CD4, FOXP3, and IL2RA, but further distinguished by cluster‐specific expression of naive markers (LEF1, SELL), follicular regulatory T cell markers (IL10, CXCR5), and suppressive Treg markers (CTLA4, CCR8). CD4_C6_IL17A and CD8_C8_IL17A clusters highly expressed classic markers of Th17 cells. Notably, several T cell clusters showed distinct TME distribution patterns (Figure 3G); naive T cells (CD8_C1_LEF1, CD4_C1_CCR7) were primarily enriched in the IE subtype; exhausted T cells (CD8_C9_HAVCR2, CD4_C5_CXCL13) were predominantly found in the IS subtype, while activated T cells and tissue memory T cells were mainly found in the IA subtype (CD8_C3_GZMH, CD8_C6_GNLY; Figure 3G).

In summary, by integrating bulk and single‐cell transcriptomic data, we discovered that IE was linked to activated stromal cells and angiogenesis, potentially resulting in an immune‐exclusive microenvironment. IS was correlated with the infiltration of bone marrow cells, such as neutrophils, activated mast cells, and monocytes, which could contribute to an immunosuppressive TME and resistance to ICB.^[^ ^18^ ^]^ IA was associated with infiltration of T‐NK cells and their antitumor function.

Interestingly, our study revealed that IA was more frequently observed in the early AJCC stage and had the highest TME score, while IE was biased toward the late stage and associated with worse survival outcomes. These findings suggest a potential evolutionary relationship among the three TME subtypes (Figure S2B–H, Supporting Information). To investigate this further, pseudo‐time analysis was conducted using the monocle3 package on a comprehensive signature matrix derived from bulk transcriptomic data from the ACRG, TCGA‐STAD, and GSE84437 cohorts. Remarkably, a clear trend was observed across the three TME subtypes, with an apparent evolutionary order from IA to IS, and then to, i.e., This order can be rationalized by insights into the aging microenvironment and tissue damage repair processes^[^ ^19^ ^]^ (Figure 3H–J). This potential evolutionary relationship suggests that it might be possible to reverse IE or IS to IA by targeting important biomarkers or key pathways associated with IE and IS, which could enhance immunotherapy sensitivity.

### 2.4 The Upregulation of IL‐1/IL‐1R1 Signaling May Cause TME Immunosuppression and Resistance to Immunotherapy

**Figure 4:**
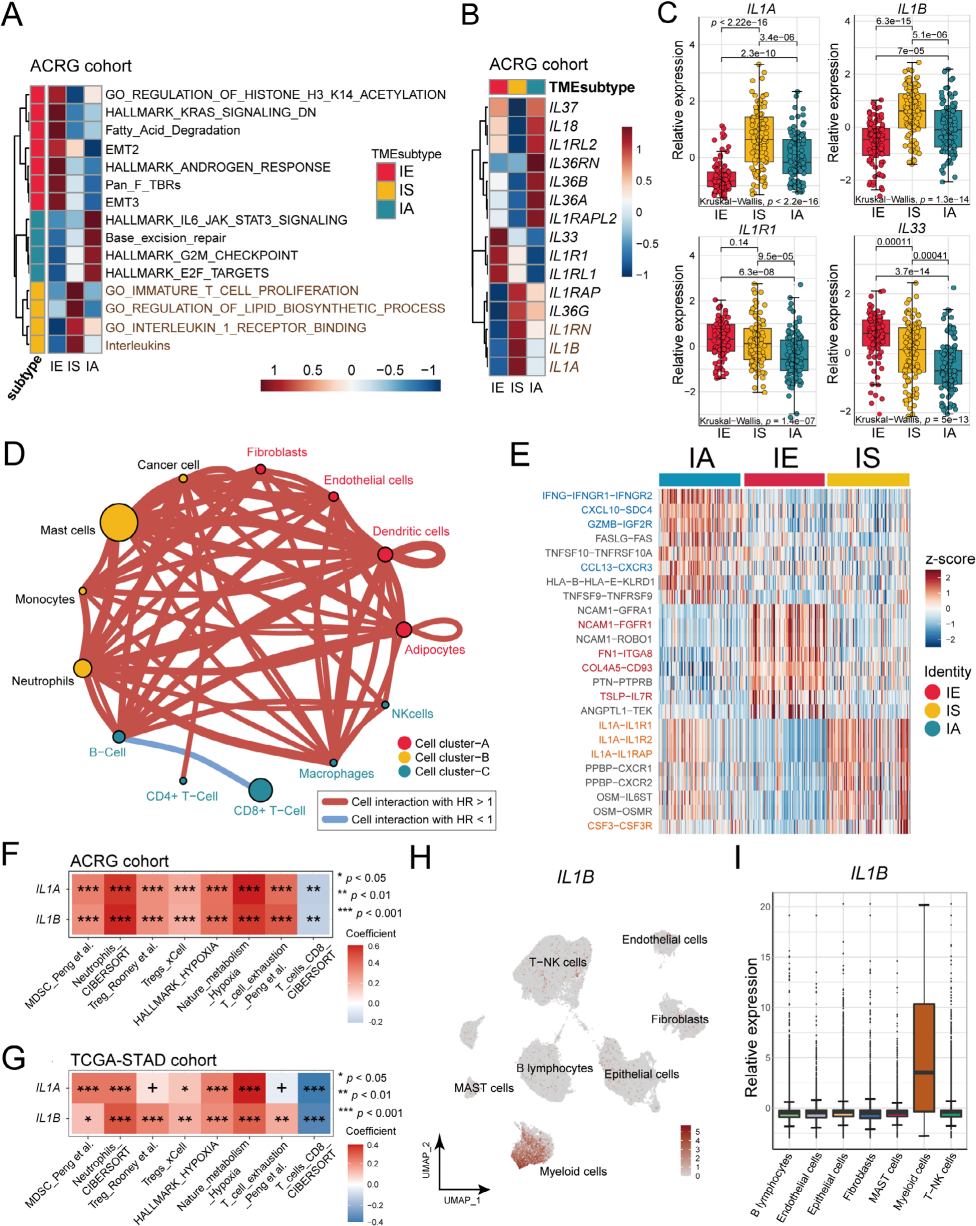
The relationship between TME subtypes and IL‐1/IL‐1R1 and other biological characteristics. A) Pathways enriched in each TME subtype represented on heatmap in ACRG cohort. B) DEGs involved in interleukin signaling among three TME subtypes in ACRG cohort. C) Differences in *IL1‐A*, *IL1‐B*, *IL1‐R1* and *IL‐33* expression among three TME subtypes in ACRG cohort. *p* values were calculated using the Mann–Whitney test for comparison between two groups, and the Kruskal‐Wallis test was used to calculate *p* values for comparisons of more than two groups. D) Differential cell‐cell interaction network of IS subtype compared to IE and IA. Different circles represent different cell types colored according to TME subtypes. The circle sizes represent the statistical significance of each cell types. Edges are weighted by the cell‐cell interactions of the connective cell types. E) Differential ligand‐receptor interactions among three TME subtypes represented on heatmap. F,G) Correlation heatmaps showing the relationship between *IL1A/B* expression and immunosuppression‐related signatures in the ACRG (F) and TCGA‐STAD cohorts (G). The correlation coefficient was computed using Spearman analysis. H,I) The expression of *IL1‐B* in the seven basal cell clusters by UMAP plot (H) and box plot (I). In box plots, *p* values were calculated using the Mann–Whitney test for comparisons between two groups. In the tumor growth curve graph, the two‐way ANOVA analysis method was used to compare the differences in tumor size between the two groups. Error bars represent the mean ± SD; ns, no significance; ^*^, *p <* 0.05; ^**^, *p* < 0.01; ^***^, *p* < 0.001; ^****^, *p* < 0.0001.

To decode the tumor immunity, metabolism, biological processes, intrinsic signaling pathway regulation, and cell‐cell interactions and identify molecular characteristics of IE and IS subtypes, we integrated gene signatures and ligand‐receptors.^[^ ^20^ ^]^ In this study, several key findings emerged. We observed significant upregulation of the pan‐fibroblast TGF‐β response signature^[^ ^11^ ^]^ (Pan‐F‐TBRS), EMT, fatty acid degradation, H3K14 acetylation, and androgen response in the IE subtype. Conversely, the IA subtype exhibited upregulation of IL‐6/JAK/STAT signaling, base excision repair, G2 M checkpoint, and E2F targets. Additionally, we observed enhanced immature T cell proliferation, lipid biosynthetic process, and IL‐1/IL‐1R1 interaction in the IS subtype (**Figure** **4**A). Numerous studies have highlighted the association between the upregulation of IL‐1/IL‐1R1 and their downstream signaling pathway with immunosuppression and resistance to immunotherapy.^[^ ^21^ ^]^ Specifically, Zhang et al. demonstrated that high expression of IL‐1R1 led to resistance to both immunotherapy and adjuvant chemotherapy in gastric cancer.^[^ ^21a^ ^]^ These findings underscore the significance of IL‐1/IL‐1R1 signaling in treatment resistance mechanisms.

Subsequently, our focus shifted toward investigating the relevance of the IL‐1/IL‐1R1 interaction to the TME. We conducted an analysis of gene expression pertaining to the IL‐1 family ligands and receptors, which revealed that the crucial ligands IL‐1α/β exhibited high expression levels in the IS subtype (the respective *p* values are provided in the results; Figure 4B,C). These findings were further validated using data from the TCGA‐STAD cohort (Figure S4C–E, Supporting Information). Moreover, our analysis of cell‐cell interactions indicated that IS exhibited a greater number of malignant communications compared to IE and IA (Figure 4D; Table S4, Supporting Information). Furthermore, examination of ligand‐receptor interaction networks highlighted IL‐1/IL‐1R1 as the primary ligand‐receptor interaction within the IS (Figure 4E; Figure S4F and Table S5, Supporting Information). These results strongly suggested that the upregulation of IL‐1/IL‐1R1 interaction in IS may be closely associated with the biological characteristics of this particular subtype.

Previous studies have revealed that IL‐1 can mobilize and recruit the MDSC and neutrophil to shape an immunosuppressive microenvironment, which might repress the T cell‐mediated antitumor function.^[^ ^18^, ^21^, ^22^ ^]^ Correspondingly, correlation analysis showed that IL‐1 expression was positively correlated with several immunosuppressive cells’ infiltration, hypoxia, and T cells exhaustion while negatively correlated with the infiltration level of CD8^+^ T cells in gastric cancer (Figure 4F,G). These results further highlighted the crucial role of IL‐1 in regulating the phenotype of IS. Interestingly, single‐cell RNA sequencing data analysis confirmed that IL1B was highly expressed in myeloid cells (Figure 4H,I), hinting that there may be a positive feedback loop of IL‐1 release and chemotactic recruitment of myeloid cells in the IS subtype.

Concurrently, the IL‐1 receptor gene, IL‐1R1, exhibited elevated expression levels in fibroblasts (Figure S4G,H, Supporting Information). This observation suggests that specific myeloid cells expressing IL‐1 may activate fibroblasts, partially explaining the dynamic evolution of TME subtypes. In addition, we noted significantly higher expression of IL‐33, another ligand from the IL‐1 family, in the IE subtype (Figure 4B,C). Increased expression of IL‐33 has been associated with immunosuppression, reduced cytotoxicity of CD8^+^ T cells, and impaired antitumor function.^[^ ^23^ ^]^ A recent study suggested that IL‐33 can facilitate the fibroblast‐to‐myofibroblast transition through M2 macrophages, thereby promoting cancer progression and metastasis.^[^ ^24^ ^]^ Notably, the IE subtype exhibited abundant expression of IL‐33, along with an abundance of the aforementioned cell types.

Building on these findings, we further explored the TME subtypes in an in vivo mouse model to better understand the cellular and molecular dynamics associated with tumor progression and immunotherapy response. In mouse experiments, three murine gastric cancer cell lines, YTN2, YTN5, and YTN16^[^ ^25^ ^]^ were utilized for subcutaneous tumor development. We aimed to assess the impact of distinct transcriptional profiles and TME classifications on tumor behavior and therapeutic outcomes. Although these three cell lines were established by same experimental method flow and share many similarities, they exhibit many differences in terms of formation rate, growth speed, and the transcriptional characteristics of subcutaneous tumors.

**Figure 5:**
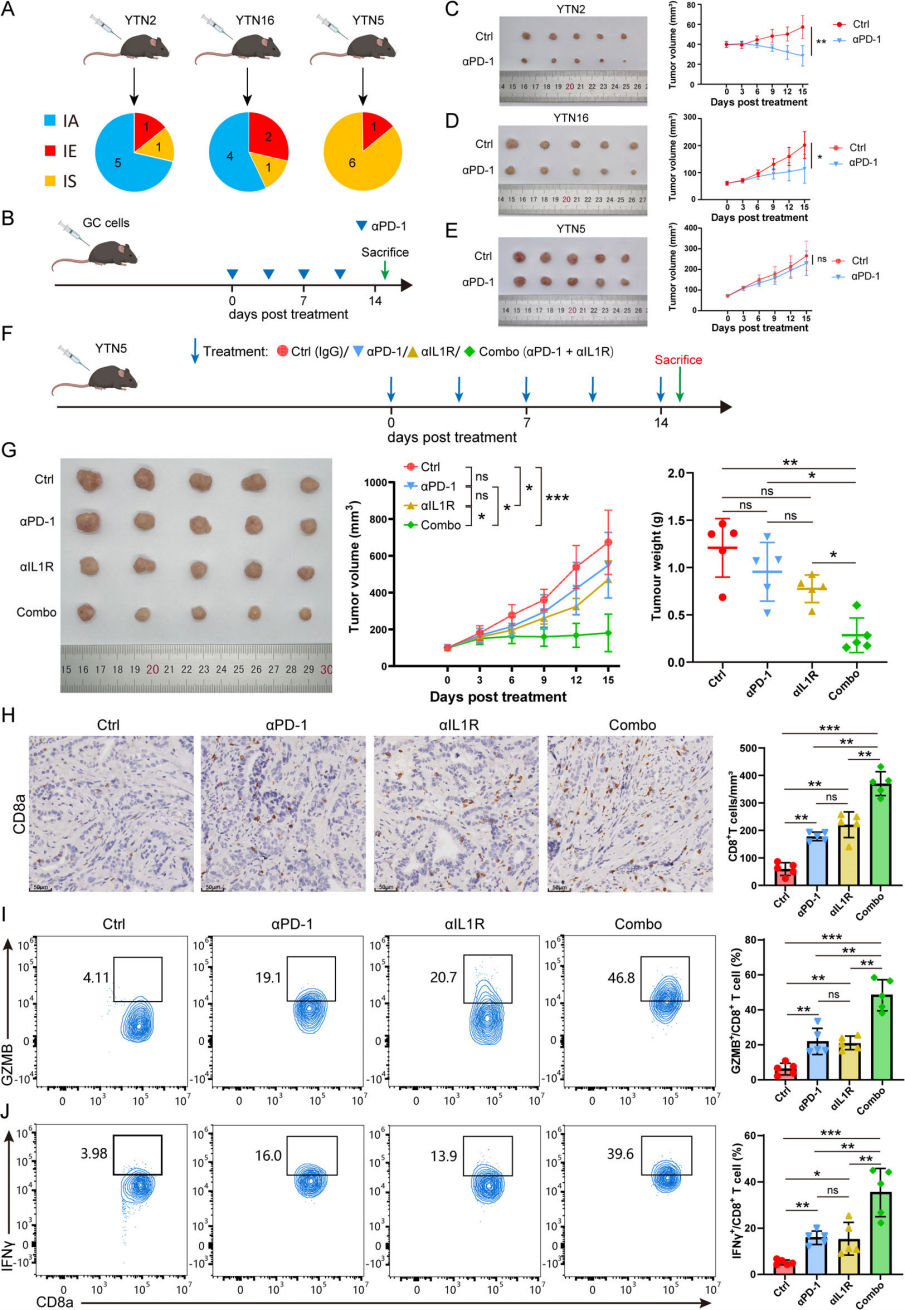
A) Pie charts displaying the distribution of different TME subtypes in the three types of subcutaneous tumors (*n =* 7 per group). B) The flow chart of treatment protocol. C–E) Images and growth curves of subcutaneous tumors formed by three cell lines (*n =* 5 per group). F) The flow chart of treatment protocol. G) Images and growth curves of subcutaneous tumors formed by three cell lines (*n =* 5 per group). H) Representative IHC images showing differential CD8^+^ T cell infiltration in subcutaneous tumors of the four groups and quantification of positive cell density (right panel; *n =* 5 per group). I,J) Representative flow cytometer results showing cytokine expression levels of CD8^+^ T cell of the four groups and quantification of positive subpopulation (right panel; *n =* 5 per group) In box plots, *p* values were calculated using the Mann–Whitney test for comparisons between two groups. In the tumor growth curve graph, the two‐way ANOVA analysis method was used to compare the differences in tumor size between the two groups. Error bars represent the mean ± SD; ns, no significance; ^*^, *p <* 0.05; ^**^, *p* < 0.01; ^***^, *p* < 0.001; ^****^, *p* < 0.0001.

When applied TMEclassifier to the RNA‐seq data of tumors formed by the three cell lines, we found that the YTN2 and YTN16 tumors possessed IA‐like TME (5/7 and 4/7, respectively), while tumors were predominantly categorized as IS subtype in YTN5 (6/7; **Figure** **5**A). In the immunotherapeutic experiment, only the YTN5 tumor showed resistance to the anti‐PD‐1 antibody, which was consistent with our hypothesis (Figure 5B–E). For the IS‐subtype YTN5 tumor, we applied the IL1R inhibitor and found that it can reverse the resistance of YTN5 tumors to immunotherapy, significantly enhancing the tumor‐killing effect of PD‐1 blockade (Figure 5F,G). Immunohistochemistry and flow cytometry experiments (Figure 5H–J; Figure S4I, Supporting Information) demonstrated that targeting IL‐1/IL‐1R1 signaling can increase the infiltration abundance of CD8^+^ T cells and M1‐type macrophages in the TME and activate cytotoxic T lymphocytes (CTLs) to secrete cytotoxic factors, shifting the TME toward a hot tumor phenotype.

### 2.5 Validation of TME Subtyping as a Predictive Biomarker for Immunotherapy Response in Gastric Cancer and Other Tumor Types

**Figure 6:**
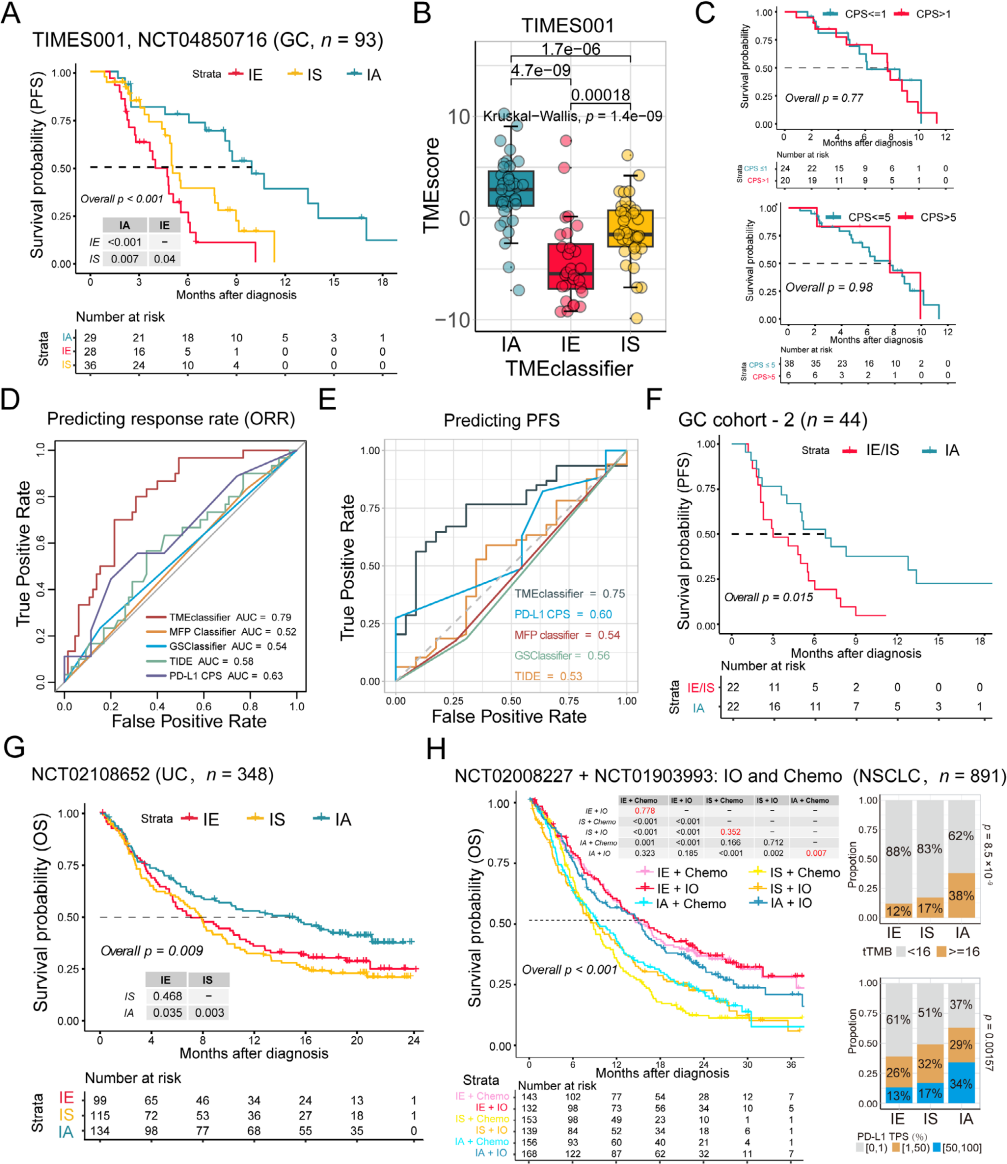
Validation of TME Typing as a Predictive Biomarker for Immunotherapy Response in Gastric Cancer and Other Tumor Types. A) Kaplan–Meier curves of progression‐free survival (PFS) by TME subtypes in our TIMES001 prospective clinical trial (NCT04850716, n = 93). B) Box plots of difference in TMEscore among TME subtypes in TIMES001 clinical trial. C) Kaplan–Meier curves of PFS by CPS (top: cutoff = 1; bottom: cutoff = 5) in the TIMES001 clinical trial. D) Receiver operating characteristic (ROC) analyses showing the comparison of TMEclassifier, MFP classifier, GSClassifier, TIDE, and PD‐L1 CPS for predicting ICBs response in the TIMES001 clinical trial. E) Receiver operating characteristic analyses showing the comparison of TMEclassifier, MFP classifier, GSClassifier, TIDE, and PD‐L1 CPS for predicting 8 months or more PFS (bottom) in the TIMES001 clinical trial. F) Kaplan–Meier curves of progression‐free survival (PFS) by TME subtypes in the GC NanoString cohort (GC IO cohort 2, n = 44). G) Kaplan–Meier curves of OS by TME subtypes in the IMvigor210 cohort (NCT02108652, n = 348). H) Left panel: Kaplan–Meier curves of OS by TME subtypes with different treatments of patients from the OAK and POPLAR combined cohort (NCT02008227 and NCT01903993, n = 891). Each group was color‐coded as follows: IE with chemotherapy, pink; IS with chemotherapy, yellow; IA with chemotherapy, wathet; IE with immunotherapy, red; IS with immunotherapy, orange and IA with immunotherapy, blue. Right panels: Proportion of tTMB level (upper) and PD‐L1 TPS (bottom) among three TME subtypes of patients in OAK and POPLAR cohort. In box plots, p values were calculated using the Mann–Whitney test for comparison between two groups, and the Kruskal‐Wallis test was used to calculate p values for comparisons of more than two groups. In bar plots, the Chi‐squared test p values are displayed at the top of the figure. The log‐rank test was used to assess the statistical significance of the prognostic differences among the subtypes in the survival analyses above. CR, complete response; PR, partial response; SD, stable disease; PD, progressive disease; tTMB, tissue tumor mutation burden; CPS, PD‐L1 combined positive score; TPS, PD‐L1 tumor proportion score.

To validate the correlation between TME typing and immunotherapy response, we conducted a TIMES‐001 prospective observational clinical trial (NCT04850716) involving 93 gastric cancer specimens collected before immunotherapy combined with chemotherapy. By utilizing RNA‐seq data and the TMEclassifier machine learning model, we obtained remarkable results. Patients classified as IA showed significantly improved progression‐free survival (PFS) compared to those with IE and IS after receiving ICB inhibitors plus chemotherapy (*p* < 0.001; **Figure** **6**A). The IA subtype had the highest TMEscore^[^ ^8^ ^]^ (*p* < 0.001; Figure 6B), suggesting that IA subtype have the best TME immune infiltration which may render higher response rate than others.^[^ ^8^, ^9^ ^]^ However, using the conventional biomarker PD‐L1 CPS in clinical practice, there was no statistically significant difference in PFS between high and low groups, regardless of the cutoff values (*p* = 0.77 and *p* = 0.98, respectively; Figure 6C). Receiver operating characteristic (ROC) analyses indicated that the TMEclassifier outperformed PD‐L1 combined positive score (CPS), TIDE score,^[^ ^26^ ^]^ and other two Tumor classification system^[^ ^27^ ^]^ in predicting both ICBs response rate (ORR) and progression‐free survival in TIMES001 clinical trial (Figure 6D,E; Figure S5A–D, Supporting Information). Additionally, the efficacy of predicting PFS was further enhanced when the model combined the expression levels of *GZMB* and *CD274* genes (Figure S5E, Supporting Information). Furthermore, the outcomes of the decision curve analysis underscored the clinical significance of the TMEclassifier in guiding clinical decisions (Figure S5F, Supporting Information). These findings demonstrated the potential of TME classification as a reliable predictive biomarker for immunotherapy response in gastric cancer patients, also confirmed with clinical data that IS and IE immunophenotyping may be resistant from immunotherapy.

In multicenter retrospective gastric cancer immunotherapy cohort (GC cohort‐2, *n =* 44), TME typing assessment was performed on patients using NanoString assay data.^[^ ^9^ ^]^ Due to the absence of certain TME feature genes, a small number of patients (*n =* 2) were classified as IE, Therefore, we combined IE and IS patients into the IE/IS group for further analysis. Gastric cancer patients with the IA phenotype displayed a superior prognosis (*p* = 0.015; Figure 6F) and a higher response rate compared to IE/IS in both the NanoString cohort^13^ and another gastric cancer immunotherapy cohort^[^ ^12^ ^]^ (NCT02589496) (21% vs 46% and 10% vs 18% vs 44%, respectively; Figure S6G, Supporting Information). TMEscore was significantly higher in IA subtype (*p* = 1.4×10^−9^ and *p* = 1.3×10^−5^, respectively; Figure 6B; Figure S5G, Supporting Information), consistent with our previous study, and verified TMEscore as a robust predictive biomarker of ICB response.^[^ ^8^ ^]^ We then used the median value of TMEscore as a cutoff and selected tumors with high TMEscore from the aforementioned cohorts.^[^ ^9^, ^12^ ^]^ We observed that the proportion of responders in IE or IS was lower than that in IA (37.5% vs 62.5% and 29% vs 53%, respectively; Figure S5H, Supporting Information), suggesting that combining TMEscore with TMEclassifier (to filter out patients with high TMEscore but classified as IE or IS) could optimize our preliminary work and further improve the accuracy of predicting immunotherapy efficacy.

Furthermore, to assess the applicability of the TMEclassifier across various tumor types, we gathered datasets from immunotherapy cohorts representing different cancer types. When we applied the TMEclassifier to the IMvigor210 immunotherapy cohort for urothelial carcinoma (NCT02108652, *n =* 348),^[^ ^11^ ^]^ we observed similar results in terms of response rate and prognosis after immunotherapy (refer to the results for corresponding *p* values; Figure 6G). The Molecular Functional Portrait (MFP) subtype^[^ ^27a^ ^]^ indicated that the immune‐enriched (IE) subtype may have a significant survival benefit from immunotherapy, similar to the IA TME defined by the TMEclassifier (Figure S6A,B, Supporting Information). But another classifier of the gastric cancer did not identify patients who could benefit from immunotherapy (Figure S6C,D, Supporting Information). Also, in this cohort, we again see that TMEclassifier has the highest predictive accuracy than other biomarkers^[^ ^1^, ^26^, ^27^ ^]^ in predicting patient progression‐free survival (AUC = 0.64; Figure S6E, Supporting Information). Likewise, in the hepatocellular carcinoma (HCC) immunotherapy cohorts^[^ ^28^ ^]^ of GO30140 (NCT02715531) and IMbrave150 (NCT03434379), IS subtypes identified by the TMEclassifier have a significantly worse prognosis than IE and IA subtypes (*p* < 0.001; Figure S6F, Supporting Information), while the IE and IA subtypes had a similar survival prognosis. The MFP subtype indicates that high immune infiltration or concomitant fibrosis responds better to therapy (Figure S6G, Supporting Information). In this cohort, only two subtypes were identified by the GSClassifer,^[^ ^27b^ ^]^ and although there was a survival difference between these two subtypes of patients (Figure S6H, Supporting Information), this suggests that the model may suffer from instability when dealing different data or cancer types. Consistently, these data suggest that the immunosuppressive microenvironment may be more critical in mediating immunotherapy resistance in HCC. ROC analysis indicated that TMEclassifier has the highest predictive accuracy than other biomarkes (TIDE, CD274 gene expression, MFP subtypes and GSClassifier) in predicting progression‐free survival (AUC = 0.69; Figure S6K, Supporting Information).

In the non‐small cell lung cancer (NSCLC, NCT04879316, *n =* 55),^[^ ^29^ ^]^ the results showed that IA phenotype was associated with longer overall survival and a higher response rate to immunotherapy compared to IE/IS tumors (*p* = 0.023 and *p* = 0.01, respectively; Figure S7A, Supporting Information). Interestingly, regardless of treatment with chemotherapy or immunotherapy, IE exhibited better survival outcomes, while IS was associated with the worst prognosis after second‐line immunotherapy in the OAK and POPLAR NSCLC combined cohort (both *p* < 0.001, Figure S7B,C, Supporting Information).^[^ ^7a^ ^]^ This suggests that the role of the TME varies across different cancers. Notably, patients with the IA subtype of NSCLC showed a significantly better prognosis when treated with immunotherapy compared to chemotherapy (*p* = 0.007; Figure 6H; Figure S7D, Supporting Information), providing strong support for the preference of immunotherapy in IA patients. Surprisingly, when considering both the chemotherapy and immunotherapy groups together, there was no statistically significant difference in the survival of IE or IS patients when comparing immunotherapy to chemotherapy (A: *p* = 0.778, and B: *p* = 0.352; Figure 6H; Figure S7E,F, Supporting Information). Additionally, IA tumors exhibited higher infiltration of CD8^+^ T cells and M1‐macrophages (Figure S7G, Supporting Information). On the other hand, patients were divided into three groups (low, medium, and high) based on PD‐L1 TPS levels. Among patients who received chemotherapy or immunotherapy, there was no significant difference in prognosis among the three groups (Figure S7H, Supporting Information). This indicated that the PD‐L1 TPS might be inappropriate for guiding the selection of drug regimens in this second‐line treatment cohort, whereas the TME classification model was a better indicator. In this cohort, we again observed that IE typing identified by MFP can significantly benefit from immunotherapy compared to chemotherapy (Figure S8A, Supporting Information), and these results again demonstrate the importance of identifying the tumour microenvironment cluster. At the same time, we observed that the GSClassifier exhibited an unstable patient typing component and that an unexplained relationship between phenotype and treatment response emerged (Figure S8B–D, Supporting Information). The survival analysis result of TCGA pan‐cancer (*n =* 7657; Figure S8E, Supporting Information) was similar to the OAK and POPLAR NSCLC combined cohort, suggesting that the IS subtype was a generally poor prognosis subtype and harbor resistance capacity to both immunotherapy and chemotherapy (Figures 2G and 6G). However, IA had the highest expression of immune checkpoint genes and CD8^+^ T cell‐relevant genes in the pan‐cancer landscape (Figure S8F, Supporting Information), suggesting a potential augmentation of the ICB response. These results indicate that although there may not be a statistical difference in prognosis between IE and IA in some cohorts, patients with IA tumors are still the most likely to benefit from immunotherapy.

### 2.6 Summary of Distinct TME Subtypes and Their Prognostic and Immunotherapeutic Relevance in Cancer

**Figure 7:**
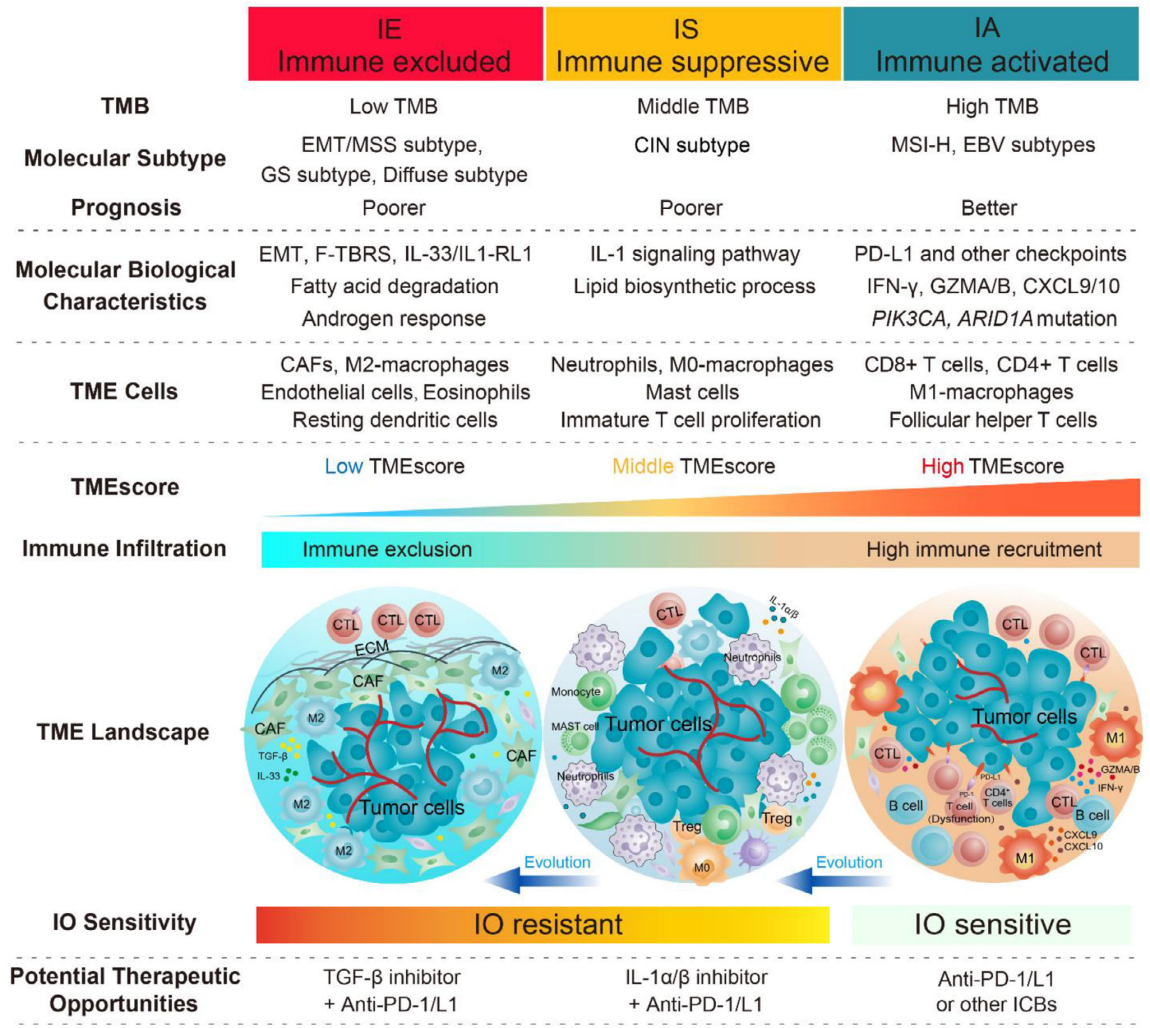
Graphical abstract for comprehensive characterization of TME subtypes of gastric cancer. The three TME subtypes displayed significantly distinct cellular and molecular features. TMB: tumor mutational burden; F‐TBRS, pan‐fibroblast TGF‐β response signature; CAFs, cancer‐associated fibroblasts; CTL, cytotoxic T lymphocyte.

Through the aforementioned exploration, we have classified cancer into three distinct TME subtypes and have identified differences among these subtypes in various characteristics (**Figure** **7**). Here, we provide a summary as follows: i) Molecular subtype: IE subtype showed a strong association with EMT/MSS, GS, and diffuse subtypes; IS and CIN subtypes demonstrated relevance; IA subtype was primarily associated with MSI‐H and EBV subtypes. ii) Molecular biological characteristics: IE subtype exhibited upregulation of EMT, pan‐F‐TBRS, IL‐33/IL‐1RL1, fatty acid degradation, and androgen response signaling pathways; IS subtype presented an increase in IL‐1/IL‐1R1 interaction and lipid biosynthetic process; IA subtype expressed more immune checkpoint and immune‐activated related genes and harbored more *PIK3CA* and *ARID1A* mutations. iii) High infiltrating TME cells: IE subtype demonstrated high infiltration of CAFs, endothelial cells, M2‐macrophages, eosinophils, and resting dendritic cells; IS subtype showed an abundance of neutrophils, M0‐macrophages, and mast cells; IA subtype exhibited high levels of CD8^+^ T cells, M1 macrophages, activated memory CD4^+^ T cells, and follicular helper T cells. iv) Gradual Increase in TMB, TMEscore, and Immune Infiltration: From IE to IS, and then to IA subtypes, there was a gradual increase in tumor mutational burden (TMB), TMEscore, and immune infiltration. v) Prognostic and Immunotherapeutic Relevance: IE and IS subtypes were associated with a poorer prognosis and displayed resistance to immunotherapy; Patients with IA subtype exhibited better survival outcomes and were more likely to benefit from immunotherapy.

## 3 Discussion

This study aimed to develop a robust TME subtyping model for cancer and evaluate its clinical and molecular characteristics, survival outcomes, and potential for immunotherapy efficacy in gastric cancer. The researchers employed gene expression data from diverse cancer cohorts to estimate TME cell infiltration and identified three distinct patterns: Immune Exclusive (IE), Immune Suppressive (IS), and Immune Activated (IA). To establish a practical and dependable TME subtype classification model, machine learning algorithms were employed, and its accuracy and reproducibility were rigorously assessed. Subsequently, the TME subtypes were compared in relation to clinical features, survival outcomes, molecular characteristics, and the efficacy of immunotherapy in cancer.

Multiple previous studies have classified gastric cancer into different subtypes based on the distinct internal features of tumor cells.^[^ ^5^, ^6^ ^]^ Among these studies, the best‐known approach for gastric cancer classification is the TCGA molecular classification. Kim et al. reported that the EBV and MSI subtypes are ICB‐sensitive, whereas the GS and CIN subtypes are resistant to ICB.^[^ ^12^ ^]^ However, tumor cells often possess various driver mutations, resulting in significant heterogeneity in genomic statuses and intrinsic characteristics.^[^ ^16^, ^30^ ^]^ In our study, we focused on other cell types in the TME, which exhibit greater conservation and less heterogeneity than tumor cells across patients and cancer types.^[^ ^27a^ ^]^ The differences primarily manifest in the level of infiltration, the proportion of distinct subpopulations, and the functional status of each cell type. By comprehensively evaluating the microenvironmental conditions, we can better elucidate the underlying mechanisms of sensitivity and resistance to immunotherapy from a different perspective. This approach may also prove more valuable in developing a robust prognostic system for assessing treatment efficacy in gastric cancer and potentially other cancer types.^[^ ^4^, ^8^, ^27^, ^31^ ^]^

We observed that the IA subtype exhibited high expression levels of immune checkpoint genes and genes associated with effector CD8^+^ T cell function, indicating enhanced T cell‐mediated antitumor activity and potential sensitivity to ICB therapies. The IA subtype tumors were predominantly classified as MSI and EBV subtypes, associated with improved prognosis and response to ICB therapies. Additionally, the IA subtype tumors exhibited a higher TMB, suggesting a higher number of neoantigens and increased immune activation. In contrast, the IE subtype exhibited increased infiltration of CAFs, endothelial cells, M2‐macrophages, eosinophils, and resting dendritic cells. We also found an increasing trend in the TMEscore from IE to IS to IA, with IA showing the highest TMEscore, suggesting that the TME subtypes may serve as predictive markers for immunotherapy response in cancer.

In this study, we developed a TME‐based classification methodology and comprehensively demonstrated the multiple characteristics of three TME subtypes, as illustrated in Figure 7. To further advance research on TME in gastric cancer and other cancer types, we also created the R package TMEclassifier. This efficient tool enables us to explore clinical applicability and guide individualized precision treatment. Survival analysis demonstrated that IA tumors had the most favorable overall survival, while IE and IS tumors displayed poorer prognoses. These findings were consistent across different cohorts analyzed for TME subtyping, further supporting the prognostic value of the TME subtypes. In a previous study,^[^ ^8^ ^]^ we identified three distinct TME clusters by inferring TME infiltration and applying unsupervised clustering. However, at that time, we had not yet developed an efficient and robust methodology for TME classification. Instead, we established TMEscore^[^ ^8^ ^]^ to evaluate the overall TME, mainly based on immune and stromal‐related gene signatures. However, TMEscore did not consider the signatures of the IS subtype, nor did it explore the inherent characteristics of IS. Consequently, the IE and IS subtypes, which generally exhibited low to medium TMEscores, were grouped together as ICB‐resistant groups. Similarly, the studies of Martin‐Serrano et al.^[^ ^4^ ^]^ and Bagave et al.^[^ ^27a^ ^]^ primarily focused on immune infiltration, tumor malignancy, and fibrotic levels, but did not classify an immunosuppressive subtype. Neglecting the immunosuppressive subtype, referred to as IS in our study, would limit our comprehensive understanding of the TME.^[^ ^31^ ^]^

We developed a methodology to construct a robust and efficient tool called TMEclassifier. Unlike the complicated TME clustering process,^[^ ^8^ ^]^ TMEclassifier allows for easy classification of gastric cancer and other cancers, thereby improving the convenience and repeatability of researchers' analysis. Through comprehensive analysis, we validated two significant findings related to the TMEclassifier. First, TMEclassifier can be used for predicting the efficacy of immunotherapy. We observed higher levels of multiple biomarkers associated with positive immunotherapy response in IA. Furthermore, analysis of various immunotherapy cohorts consistently demonstrated that IA tumors were more responsive to immunotherapy compared to IE and IS tumors. The second clinical significance of the TMEclassifier is its ability to facilitate the systematic exploration of intrinsic characteristics of ICB‐resistant subtypes across multiple cohorts, thereby identifying potential therapeutic targets. Based on our current findings, we determined that IE and IS exhibit ICB resistance driven by distinct mechanisms, highlighting the importance of tailored treatment approaches for these subtypes.^[^ ^31^ ^]^

It is essential to identify key molecular biomarkers or crucial regulatory pathways associated with IE and IS subtypes in order to implement targeted interventions to improve the TME status and potentially reverse IE/IS to IA. IE is characterized by stromal activation and immune exclusion, while IS is associated with immunosuppression. Targeting stromal components such as TGF‐β may be a viable option for enhancing the drug sensitivity of IE.^[^ ^11^ ^]^ The situation with IS was more complex. Despite having a moderate TME score and a moderate Objective Response Rate (ORR) in several immunotherapy cohorts, IS exhibits the worst survival outcomes in cohorts with a large number of patients (e.g., OAK + POPLAR cohort, *n =* 891; GO30140 + IMbrave150 cohort, *n =* 238, TCGA pan‐cancer cohort, *n =* 7657). Additionally, adjuvant therapy (chemotherapy and/or radiotherapy) was found to significantly improve the overall survival of patients with IE and IA gastric cancers, but not in patients with the IS subtype in the ACRG cohort, indicating inherent resistance of IS tumors to adjuvant chemotherapy and radiotherapy. Overcoming primary drug resistance and improving the prognosis of IS may be more necessary but also more challenging than for IE.

In our observations, there was a significant upregulation of IL‐1/IL‐1R1 interaction in IS. IL‐1 was capable of recruiting myeloid cells to TME,^[^ ^18^, ^22^ ^]^ and some types of myeloid cells can secrete more IL‐1 to facilitate this feedback loop, together leading to immunosuppression in the TME. Moreover, IL‐1 can bind to IL‐1R1 on fibroblasts, activating fibroblasts and promoting their differentiation into inflammatory cancer‐associated fibroblasts (iCAFs) that release various inflammatory factors, further shaping an immunosuppressive TME.^[^ ^21^, ^32^ ^]^ Through the in vivo mouse experiments, we demonstrated that combined targeting IL‐1/IL‐1R1 could improve the TME of IS subtype tumor (YTN5), thereby significantly enhances its sensitivity to immunotherapy.

The validation across multiple datasets suggests that patients with IA can potentially benefit from immunotherapy, regardless of whether chemotherapy is administered. However, the proposed treatment strategies for IE and IS are solely based on bioinformatics analysis, and it remains unclear whether these strategies can effectively improve prognosis. To confirm our hypothesis, additional in vivo drug trials are required. Furthermore, our data indicates the possibility of a transition from IA to IS and subsequently to IE., This raises several crucial scientific questions: Is there truly an evolutionary relationship between these three TME subtypes? What are the factors driving their evolution? Does this process contribute to the formation of a drug‐resistant microenvironment? And is it reversible? The current results are insufficient to provide answers to these questions. Therefore, we plan to conduct further data analysis and experiments to gain a better understanding in the future.

In conclusion, this study successfully developed a robust TME subtyping model for cancer using gene expression profiles, providing a comprehensive characterization of three distinct TME subtypes and enhancing the understanding of the TME in cancer. The TME subtypes demonstrated distinct clinical and molecular characteristics, survival outcomes, and potential implications for immunotherapy response. These findings offer valuable insights into the heterogeneity of the TME and underscore the importance of considering the TME landscape in cancer prognosis and treatment decision‐making. The developed TME subtyping model holds promise for guiding personalized therapeutic strategies, such as immunotherapy. Furthermore, this efficient tool facilitates the application of our TME‐based classification system, which holds significant potential for offering valuable insights in scientific research and clinical practice.

## 4 Experimental Section

### Prospective Clinical Trial and Tumor Sample RNA Sequencing

The team conducted a prospective observational clinical trial for gastric cancer (NCT04850716). The trial protocol received approval from the Institutional Review Board and Human Ethics Committee of Nanfang Hospital, Southern Medical University, and all patients provided written informed consent prior to enrollment. A total of 93 patients with histologically or cytologically confirmed advanced gastric cancer were enrolled from five clinical centers. Formalin‐fixed paraffin‐embedded or fresh‐frozen tumor tissue samples were collected from these 93 patients at baseline, before initiating ICB treatment.

For RNA‐Seq analysis, tissue samples were collected and subjected to RNA extraction using the RNeasy Mini Kit from QIAGEN. Library construction and sequencing were performed by Shanghai Biotechnology Corporation. Strand‐specific libraries were prepared using the TruSeq Stranded Total RNA Sample Preparation kit from Illumina, following the manufacturer's instructions. Purified libraries were quantified using the Qubit 2.0 Fluorometer from Life Technologies and validated using the Agilent 2100 bioanalyzer from Agilent Technologies to confirm the insert size and calculate the molar concentration. The samples were sequenced on the Illumina NovaSeq 6000, generating 150 bp paired‐end reads. After filtering low‐quality reads, the raw FASTQ files were aligned to the GRCh37 reference genome using the Hisat2 aligner. The aligned fragments were then counted and annotated using HTSeq v0.6.0 and a GENCODE v19 annotation file, respectively.

### Animal Experiments

Male C57BL/6 mice (6–7 weeks of age) were purchased from the Experimental Animal Center of Southern Medical University. The mice were housed under specific pathogen‐free conditions, provided with standard food, given free access to purified water, and on a 12:12 light/dark cycle. The temperature was kept ≈22 °C and humidity ≈45%. Animal care and experiments were conducted according to the requirements of the Animal Ethics Committee of Nanfang Hospital, Southern Medical University.

The YTN2, YTN5 and YTN16, three subclones cell lines derived from chemically induced gastric cancers of C57BL/6 mice,^[^ ^25^ ^]^ were kindly provided by professor Sachiyo Nomura. And the morphological, in vivo and in vitro growth characteristics, as well as transcriptomic features, of these three cell lines are different from each other.

An inoculum of 7.5×10^6^ the above‐mentioned cells (in 100 µL of sterile PBS) was injected subcutaneously on the flank of three groups of mice respectively. Mice were randomized into a control group (Ctrl) or a treatment group when the average tumor volume reached ≈40 mm^3^ (YTN2 tumor) or 70–80 mm^3^ (YTN5 and YTN16 tumor). In the treatment group, the mice were intraperitoneally (I.P.) injected with 10mg k^−1^g anti‐PD‐1 (BioXcell) twice a week. For the control group, 200 µg of IgG was I.P. injected as a negative control. And in the Combination therapy experiment, YTN5 tumor‐bearing mice were randomized into four groups when the average tumor volume reached ≈100 mm^3^. Then, 200 µg of IgG, 10mg kg^−1^ anti‐PD‐1 (BioXcell), 20mg kg^−1^ anti‐IL‐1R (BioXcell) or 10mg kg^−1^ anti‐PD‐1 plus 20mg kg^−1^ anti‐IL‐1R were I.P. injected to the mice of the four groups (Ctrl, αPD‐1, αIL1R and Combo) respectively. On the 15th day post‐treatment, or if the mice exhibit adverse reactions, such as a significant weight loss, the mice will be euthanized. Tumor growth was monitored every 3 days with calipers, and tumor volume was calculated by the formula L X W^2^ X 0.5, where L and W represent length and width, respectively.

### Single‐Cell Suspension Preparation and Flow Cytometry

Fresh tumor tissues were minced into fragments and enzymatically dissociated into single‐cell suspensions using a Miltenyi Biotec mouse Tumor Dissociation Kit, strictly adhering to the manufacturer's protocol. The suspensions were filtered through a 70‐µm cell strainer to remove undigested debris. Lymphocytes were isolated by Percoll density gradient centrifugation. Purified cells were activated with a cocktail of phorbol 12‐myristate 13‐acetate (PMA, 50 ng mL^−1^), ionomycin (1 µg mL^−1^), and brefeldin A (BFA, 10 µg mL^−1^) for 5 h to stimulate cytokine secretion in CD8⁺ T cell.

Cells were stained with the Zombie Aqua Fixable Viability Kit (BioLegend) to exclude dead cells and blocked with anti‐CD16/32 antibody to minimize nonspecific binding. Surface markers were labeled with the following antibodies: BV650‐conjugated anti‐CD45, RB705‐conjugated anti‐CD3, and APC‐conjugated anti‐CD8a. Following surface staining, cells were fixed, permeabilized using a FIX & PERM Kit, and subsequently underwent intracellular staining with PE‐Cy7‐conjugated anti‐IFNγ and BV421‐conjugated anti‐GZMB (granzyme B). Stained cells were detected on a flow cytometer, and data were processed with FlowJo software.

### Data Sources and Acquisition Methods

Publicly available gastric cancer bulk RNA‐seq datasets (ACRG/GSE62254, GSE84437, GSE34942, GSE15459, GSE57303, GSE26253, and TCGA‐STAD) were obtained from the Gene Expression Omnibus (GEO) database (https://www.ncbi.nlm.nih.gov/geo/), and TCGA (UCSC Xena, https://gdc.xenahubs.net/). The scRNA‐seq data of gastric cancer were available in GEO under accession number GSE183904^[^ ^7b^ ^]^ and OMIX001073. Additionally, an immunotherapy cohort dataset was downloaded from the European Nucleotide Archive (Kim et al. gastric cancer cohort (NCT02589496),^[^ ^12^ ^]^ https://www.ebi.ac.uk/ena/browser/, accession number: PRJEB25780). The IMvigor210 dataset (NCT02108652)^[^ ^15^ ^]^ was obtained from the IMvigor210CoreBiologies R package. The multi‐omics data of the OAK (NCT02008227) and POPLAR (NCT01903993) cohorts of non‐small cell lung cancer^[^ ^9^ ^]^ were deposited at the European Genome‐phenome Archive (EGA, https://ega‐archive.org/, accession number: EGAS00001004343) and were available under restricted access. The GO30140 (NCT02715531) and IMbrave150 (NCT03434379) dataset of hepatocellular carcinoma was obtained from EGA (accession number: EGAS00001004343).^[^ ^28^ ^]^ The CERTIM cohort (NCT04879316) NanoString data was obtained from the supplementary data of the published research.^[^ ^29^ ^]^ And the NanoString data of the gastric cancer immunotherapy cohort was generated from the previous study.^[^ ^13^ ^]^

### Inference of Cell Fraction and Signature Score

Several computational tools (CIBERSORT, MCP‐counter, ESTIMATE, EPIC, and xCell) were integrated to estimate the infiltration of multiple cell types in the datasets mentioned above.^[^ ^10^ ^]^ The 22 cell types inferred by CIBERSORT and the fibroblast inferred by MCP‐counter for subsequent clustering were chosen. By the approach of gene set variation analysis (GSVA) algorithm,^[^ ^33^ ^]^ Gene Ontology (GO), Kyoto Encyclopedia of Genes and Genomes (KEGG), REACTOME, and HALLMARK gene sets were used to estimate the pathway enrichment scores for each of the samples. Other prevalent gene signature scores concerning the TME, the tumor intrinsic pathway, and metabolism were calculated for each of the samples using the Immuno‐Oncology Biological Research (IOBR) 2.0 R package.^[^ ^10^, ^34^ ^]^

### TME Subtypes Identification

The consensus clustering algorithm was applied to determine the number of clusters in the meta‐dataset and ACRG cohort to assess the stability of the discovered clusters. This procedure was performed using the ConsensusClusterPlus R package and was repeated 1000 times to ensure the stability of classification as previously elucidated.^[^ ^8^ ^]^ When unsupervised clustering analysis and set k as 3 were performed the best differentiation effect of each classification cluster was obtained, and finally three robust TME subtypes with respective typical TME infiltration patterns were classified, which was named IE, IS, and IA respectively.

### Construction of TMEclassifier

Differentially expressed genes (DEGs) of the three TME subtypes were obtained by pairwise DEG analysis in the ACRG cohort. Common genes in the three groups were removed. The dimension reduction of the remaining DEGs was performed by random forest classification algorithm, and the redundant genes were removed. Finally, 134 subtype‐specific feature genes, representing three TME subtypes respectively (A:40, B:19, C:75; Table S1, Supporting Information) were obtained. Subsequently, the samples were randomly divided into training cohort and validation cohort at a ratio of 3:1. Six machine learning algorithms (including support vector machine (SVM), random forest (RF), neural network (NNET), extreme gradient boosting (XGBoost), decision tree (DecTree), and K‐nearest neighbor (KNN)) and 134 feature genes, were applied to train the classification model in the training cohort, respectively. The probabilities of three TME subtypes predicted by the six machine learning algorithms for each tumor were averaged to calculate the ensemble probabilities of three subtypes, and each tumor would be classified as the subtype with the highest predictive probability. TMEclassifier can classify three TME subtypes based on the expression profiles of 134 TME‐related feature genes derived from bulk transcriptomic data or single cell RNAseq data with the function of R package (https://github.com/LiaoWJLab/TMEclassifier).

### Single‐Cell RNA Sequencing Data Processing

Seurat^[^ ^35^ ^]^ (version 4.0.4) was used to analyze the scRNA‐seq data of gastric cancer.^[^ ^7b^ ^]^ Specifically, the raw unique molecular identifier (UMI) matrix was processed to filter out genes detected in less than 10 cells and cells with fewer than 200 genes. Further, the numbers of gene and UMI counts for each cell were quantified and high‐quality cells with thresholds of 500 UMIs, 100 genes were preserved, and less than 25% mitochondrial gene counts to ensure that most of the heterogeneous cell types were included for downstream analyses. DoubletFinder R package^[^ ^36^ ^]^ was then applied for each sequencing library to remove potential doublets with the expected doublet rate of 9%, and cells with a double score (DF_pANN) larger than 91% quantile were filtered out. *SCTransform* was used to perform normalization in each sample and integrated them using the *harmony* function of the Harmony package.

### Single‐Cell Annotation

The gene expression values of the cells from a previous single‐cell study^[^ ^37^ ^]^ with cell annotation were used to train the prediction model using the maximum attributes dependency (MDA) algorithm, following the analysis step of the scPred R package.^[^ ^38^ ^]^ Next, the training model was applied to predict the integrated data of the 40 samples into specific cell types (epithelial cells, mast cell, T cells, B cells, myeloid cells, fibroblasts, and endothelial cells).

### TME Subtyping Using Single Cell RNAseq Data

Psedobulk analysis was applied to estimate gene expression in integrated seurat object using the function *AggregateExpression* of the Seurat R package. Following by the gene expression normalization between each sample and TME subtype gene signatures scoring process. Then, the TME subtype model was adopted to predict the TME phenotype of each sample. TMEclassifier can process single‐cell RNAseq data subtyping by importing the Seurat object (https://github.com/LiaoWJLab/TMEclassifier).

### Pseudotime Analysis

The monocle3 R package^[^ ^39^ ^]^ (version 1.2.9) was used to conduct pseudo time analysis to visualize the trajectory trend of TME subtypes using default parameters. First, a comprehensive signature matrix derived from bulk transcriptomic data in ACRG was estimated using the IOBR workflow.^[^ ^34^ ^]^ Differential express signatures of each cluster were identified and set as the input data of monocle3 using the function new_cell_*data*_set. Then, data was reduced dimensionality by the Uniform Manifold Approximation and Projection (UMAP) followed by the *learn_graph* and *plot_ccell_trajectory* function to map the differentiation and conversion of TME clusters. This analysis pipeline was applied to TCGA‐STAD and GSE84437 cohorts.

### Estimation of Cellular Interaction

The EaSIeR R package^[^ ^20^ ^]^ was adopted for the evaluation of cellular interactions based on ligand‐receptor pair and gene expression patterns. Pre‐therapy transcriptomic‐wide data were provided as input to EaSIeR, which derived patient‐specific, system‐based signatures of the TME. The paired wise Wilcoxon test was used to identify different signatures and cell‐cell interactions of each TME subtype.

### Tumor Classification Based on Molecular Functional Portrait and GSClassifier

The TME classification by the MFP platform was reported in a previous study.^[^ ^27a^ ^]^ The tumor‐immune microenvironment characteristics of each sample were deciphered by calculating the 29 functional gene expression signatures curated by the authors. Then, the unsupervised clustering was applied to classify the four TME subtypes. Detailed method and corresponding code are available in BostonGene GitHub (https://github.com/BostonGene/MFP). The above‐mentioned related analyses were performed using Python 3.11.4. The GSClassifier is an integrated R package for tumor immune subtype classification, established by Weibin Huang et al.^[^ ^27b^ ^]^ The detailed information is available in Huang's GitHub (https://github.com/huangwb8/GSClassifier).

### Quantification and Statistical Analysis

The Shapiro–Wilk normality test tested the normality of the variables. For an unpaired Student's *t*‐test estimated comparisons between two groups, statistical significance for normally distributed variables, and nonnormally distributed variables were analyzed by the Mann–Whitney U test (Wilcoxon Rank Sum test). For comparisons of more than two groups, the Kruskal–Wallis and one‐way analysis of variance tests were used for non‐parametric and parametric methods, respectively. The correlation coefficient was computed by the Spearman and distance correlation analysis. The chi‐squared test and two‐sided Fisher's exact tests were used to analyze the contingency tables. The Kaplan–Meier method was used to generate survival curves for the subgroups in each dataset, and the log‐rank (Mantel‐Cox) test was used to determine if they were statistically different. The two‐way ANOVA analysis method was used to compare the differences in tumor size in the tumor growth curve graph. All statistical analyses were conducted using R V.4.1.2 (https://www.r‐project.org/), and the *p* values were two‐sided. *p* values of less than 0.05 were considered statistically significant. The adjusted *p* value for multiple testing was calculated using the Benjamini–Hochberg correction.

### Data and Code Availability

- Original bulk RNA‐seq data of the clinical trial (NCT04850716) have been deposited at GSA‐Human (https://ngdc.cncb.ac.cn/gsa‐human/, accession number: HRA005161) and are publicly available as of the date of publication.
- The code generated for the TME classification and downstream analysis used in this manuscript can be found at https://github.com/LiaoWJLab/TMEclassifier/
- Any additional information required to reanalyze the data reported in this paper is available from the lead contact upon reasonable request.

### Ethics Statement

Samples from patients in the gastric cancer TIMES‐001 cohort of a prospective study (NCT04850716) were collected and analyzed after obtaining informed consent, which was approved by the Human Ethics Committee (NFEC‐2019‐264) of Nanfang Hospital, Southern Medical University.

### Human Tissue Samples and Subject Follow‐Up Data

Formalin‐fixed paraffin‐embedded or fresh‐frozen tumor tissue samples were collected from multiple clinical centers at baseline before initiating checkpoint immunotherapy. Tumor responses were evaluated using RECIST V.1.1 criteria. Tumor specimens from patients with advanced gastric cancer, within 90 days from the start of treatment, were processed following the protocol described by Ayers et al. Out of the 93 specimens collected from eight clinical centers (Nanfang Hospital of Southern Medical University, Sun Yat‐sen University Cancer Center, Sixth Affiliated Hospital of Sun Yat‐sen University, The First Affiliated Hospital of Xiamen University, Huizhou Municipal Central Hospital of Guangdong Province, Huizhou First Hospital of Guangdong Province, Maoming People's Hospital, and Guangxi Medical University Cancer Hospital), all 93 specimens were of sufficient quality for RNA evaluation.

## Conflict of Interest

The authors declare no conflict of interest.

## Author Contributions

D.Z., Y.Y., and W.Q. are co‐first author. D.Z. and W.L. performed conceptualization. D.Z., and W.Q. performed methodology. D.Z., Y.Y., W.Q., and H.S. curated data. D.Z., Y.Y., and W.Q. performed formal analysis and visualization. D.Z., Y.Y., W.Q., Q.M., T.G., and R.Z. performed validation. Q.M., L.J., T.G., Y. Lai, J.Z., Q.O., and Y.F. performed investigation. J.W., H.L., Y.L, H.S., M.Y., T.T., S.N., and M.S. acquired resources. J.W., H.L., and H.S. collected clinical sample. D.Z., Y.Y., W.Q., Q.M., J.Z., and Y.L. wrote original draft. D.Z., Y.Y., W.Q., Q.M., T.G., R.Z., J.W., X.H., L.J., and W.L. wrote review and editing. W.L. acquired funding. J.B., Y.L., M.Y., T.T., S.N., M.S., and W.L. performed supervision. All authors contributed to data interpretation, discussion of results, and commented on the manuscript. All authors approved the final manuscript.

## Supporting information



Supporting Information

Supporting Information

## Data Availability

The data that support the findings of this study are available on request from the corresponding author. The data are not publicly available due to privacy or ethical restrictions.;
