# Supplementary material for: Immunotyping the Tumor Microenvironment Reveals Molecular Heterogeneity for Personalized Immunotherapy in Cancer
Source: Adv Sci (Weinh). 2025 May 28;12(25):2417593. doi: 10.1002/advs.202417593 (PMC12224993; doi:10.1002/advs.202417593)
Supplement: Supplementary file 1 — Supporting Information [file ADVS-12-2417593-s001.docx]

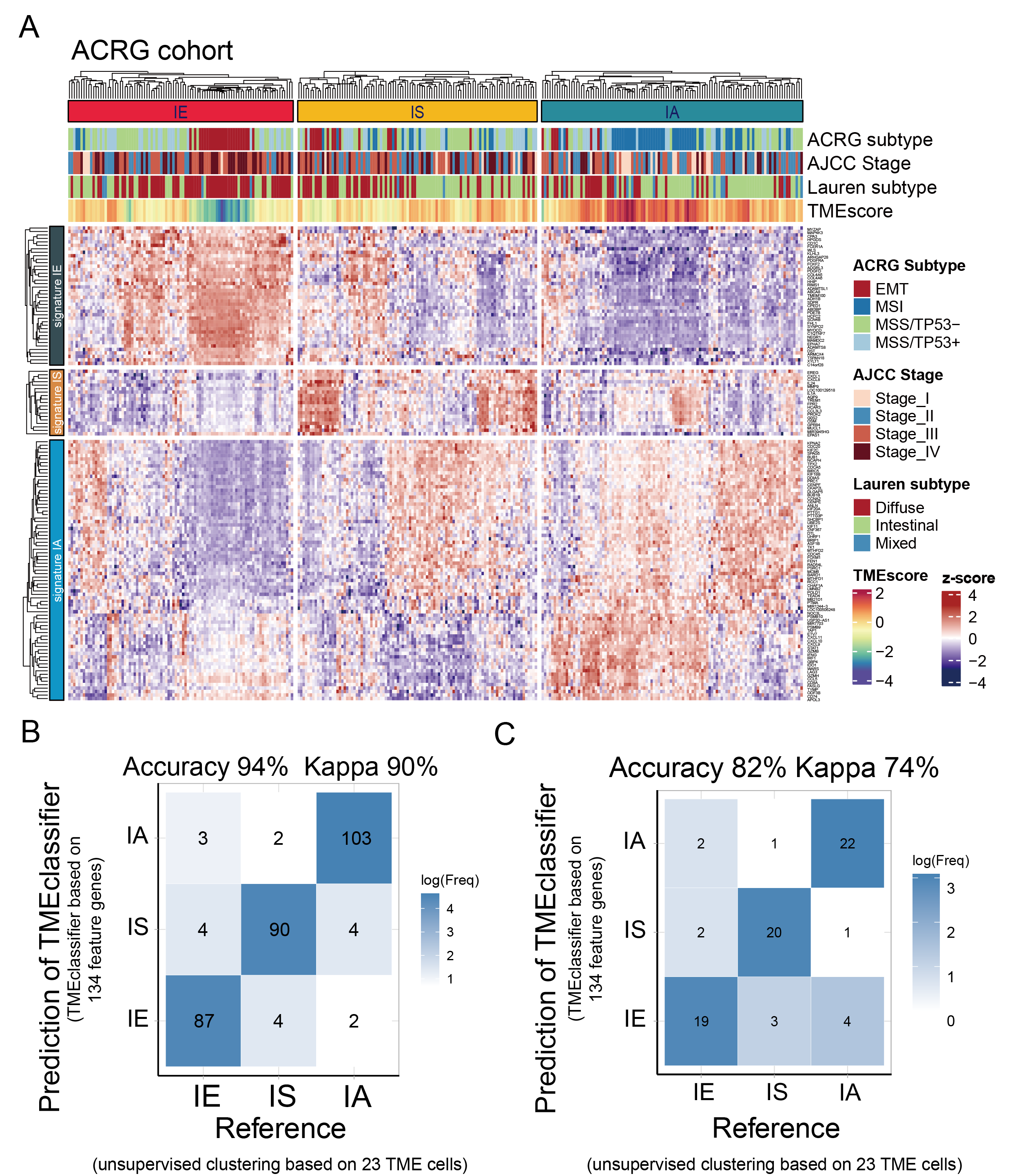
 **Figure S1**

The 134 feature genes and the classification effect of TMEclassifier.

**A)** Heatmap of three TME subtypes defined by TMEclassifier based on the expression of 134 TME related genes in ACRG cohort and correlation with clinical information.

**B and C)** Correspondence between patients classified by TMEclassifier based on 134 feature genes and unsupervised clustering based on 23 TME cells in whole ACRG cohort (**B**) and in validation cohort (**C**). The accuracy and kappa values of TMEclassifier are shown.


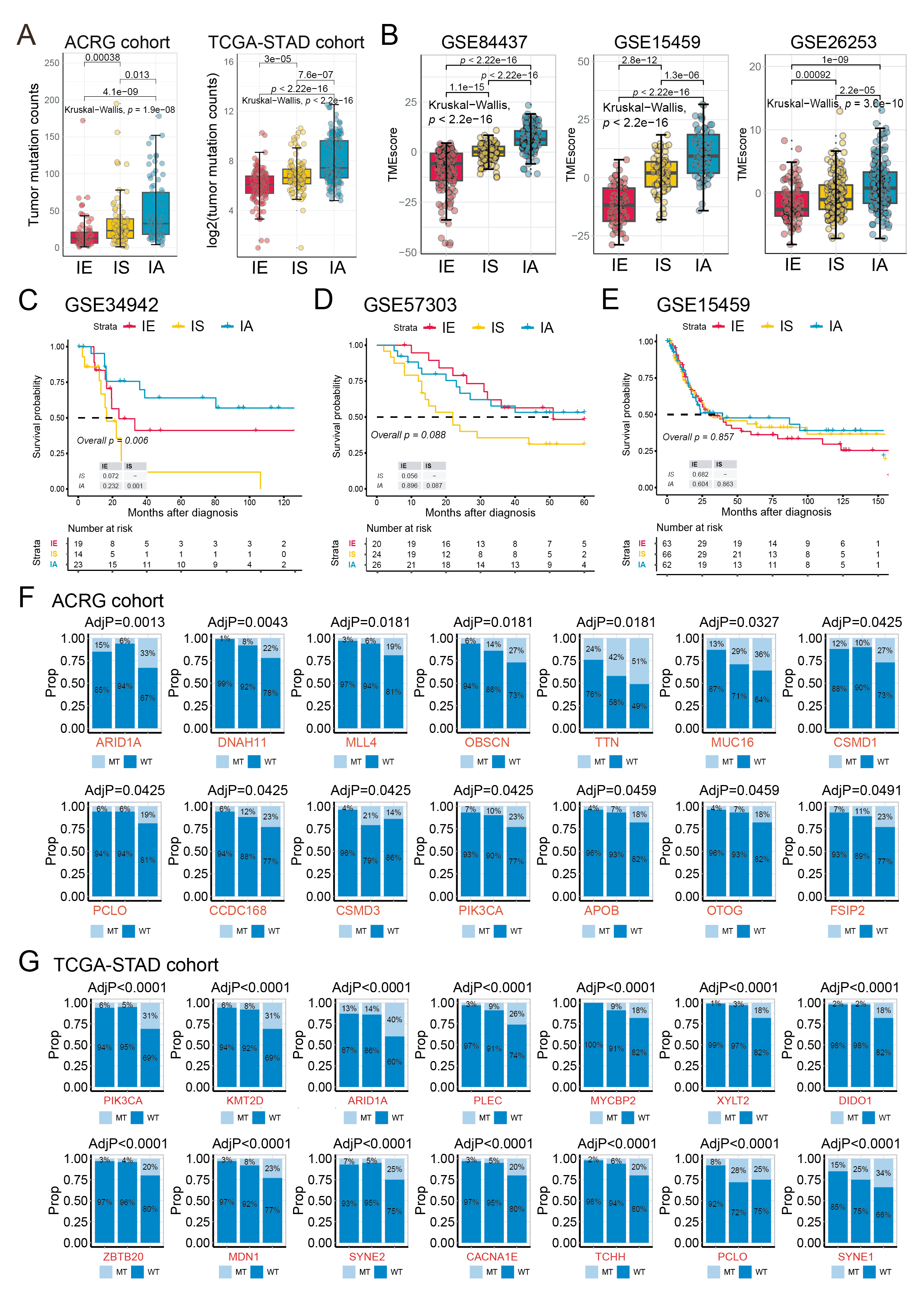


**Figure S2**

Clinical features and prognoses of TME subtypes.

**A)** Difference in tumor mutation counts of the three TME subtypes in ACGR cohort and TCGA-STAD cohort.

**B)** Difference in TMEscore of the three TME subtypes in GSE84437, GSE15459 and GSE26253 cohort.

**C-E)** Survival analysis comparing OS in GSE34942 (**C**), GSE57303 (**D**) and GSE15459 (**E**) cohorts.

In box plots, *p* values were calculated using the Mann–Whitney test for comparison between two groups, and the Kruskal-Wallis test was used to calculate *p* values for comparisons of more than two groups. The log-rank test was used to assess the statistical significance of the prognostic differences among the subtypes in the survival analyses above.

**F and G)** Proportion of different gene mutations in each TME subtype for patients in ACRG cohort (**F**) and TCGA-STAD cohort (**G**). Adjusted *p* values were evaluated by the Chi-squared test.

MT: mutation; WT: wild-type.


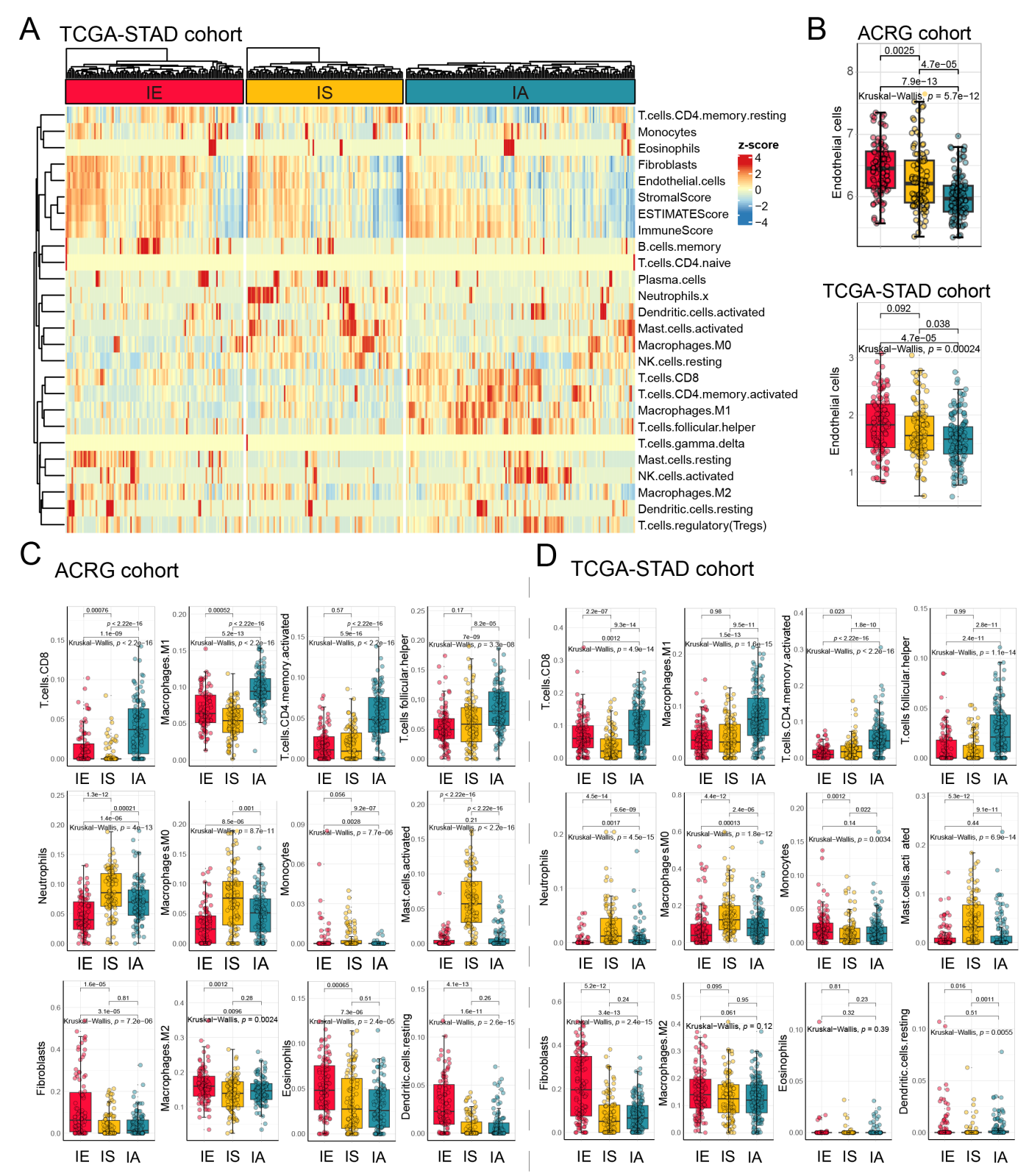


**Figure S3**

Comparison of cells’ infiltration among the three TME subtypes

**A)** Heatmap showing the infiltration levels of the 23 cell types across TME subtypes in TCGA-

STAD cohort.

**B)** Difference in infiltration of endothelial cells among three TME subtype in ACRG cohort

(upper) and TCGA-STAD cohort (bottom).

**C and D)** The comparison of cells’ infiltration among three TME subtypes in ACRG cohort (**C**)

and TCGA-STAD cohort (**D**).

In all box plots above, *p* values were calculated using the Mann–Whitney test for comparison between two groups, and the Kruskal-Wallis test was used to calculate *p* values for comparisons of more than two groups.


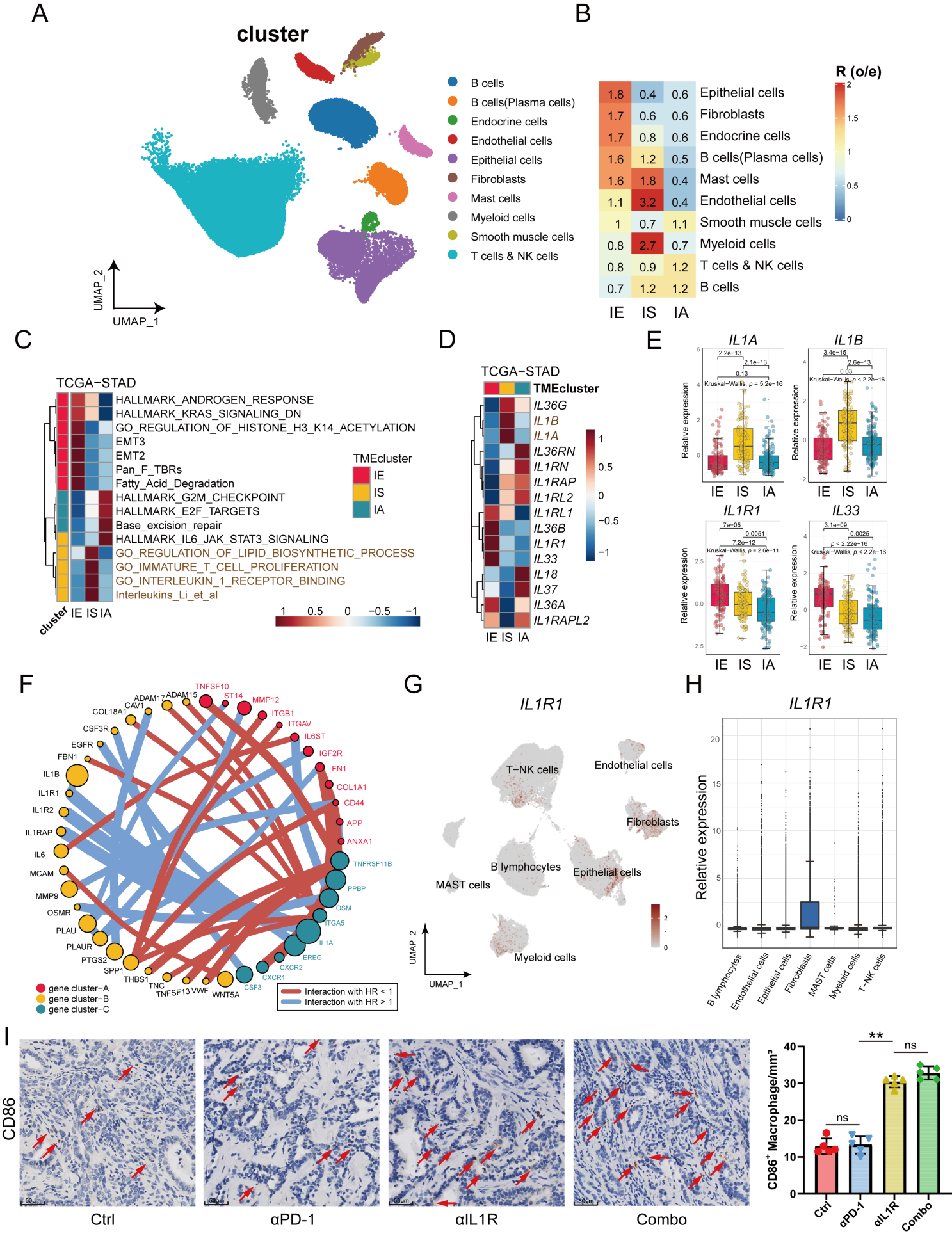


**Figure S4**

Association of TME subtypes with IL-1/IL-1R1 and some immunotherapy prediction biomarkers.

**A)** Cellular infiltration patterns of TME subtypes at single-cell resolution (OMIX001073, *n =* 10). UMAP plot annotated with ten major cell types from scRNA-seq data of ten gastric cancer samples.

**B)** Heatmap showing the preferential distribution of major cell types across different TME subtypes.

**C)** Pathways enriched in each TME subtype represented on heatmap in TCGA-STAD cohort.

**D)** DEGs involved in interleukin signaling between three TME subtypes in TCGA-STAD cohort.

**E)** Differences in *IL-1A*, *IL-1B*, *IL-1R1* and *IL-33* expression among three TME subtypes in TCGA-STAD cohort shown by box plots.

**F)** Differential ligand-receptor interaction network of IS subtype compared with IE and IA. Different circles represent different genes colored according to TME subtypes. Circle size represent the statistical significance of each gene. Edges are weighted by the statistical significance of ligand-receptor interactions compared to IE and IA.

**G** and **H**) The expression of IL1-B in the seven basal cell clusters by UMAP plot (G) and box plot (H).

**I)** Representative IHC images showing differential CD86^+^ cell infiltration in subcutaneous tumors of the four groups and quantification of positive cell density (right panel; *n* = 5 per group)

In all box plots above, *p* values were calculated using the Mann–Whitney test for comparison between two groups, and the Kruskal-Wallis test was used to calculate *p* values for comparisons of more than two groups.

**
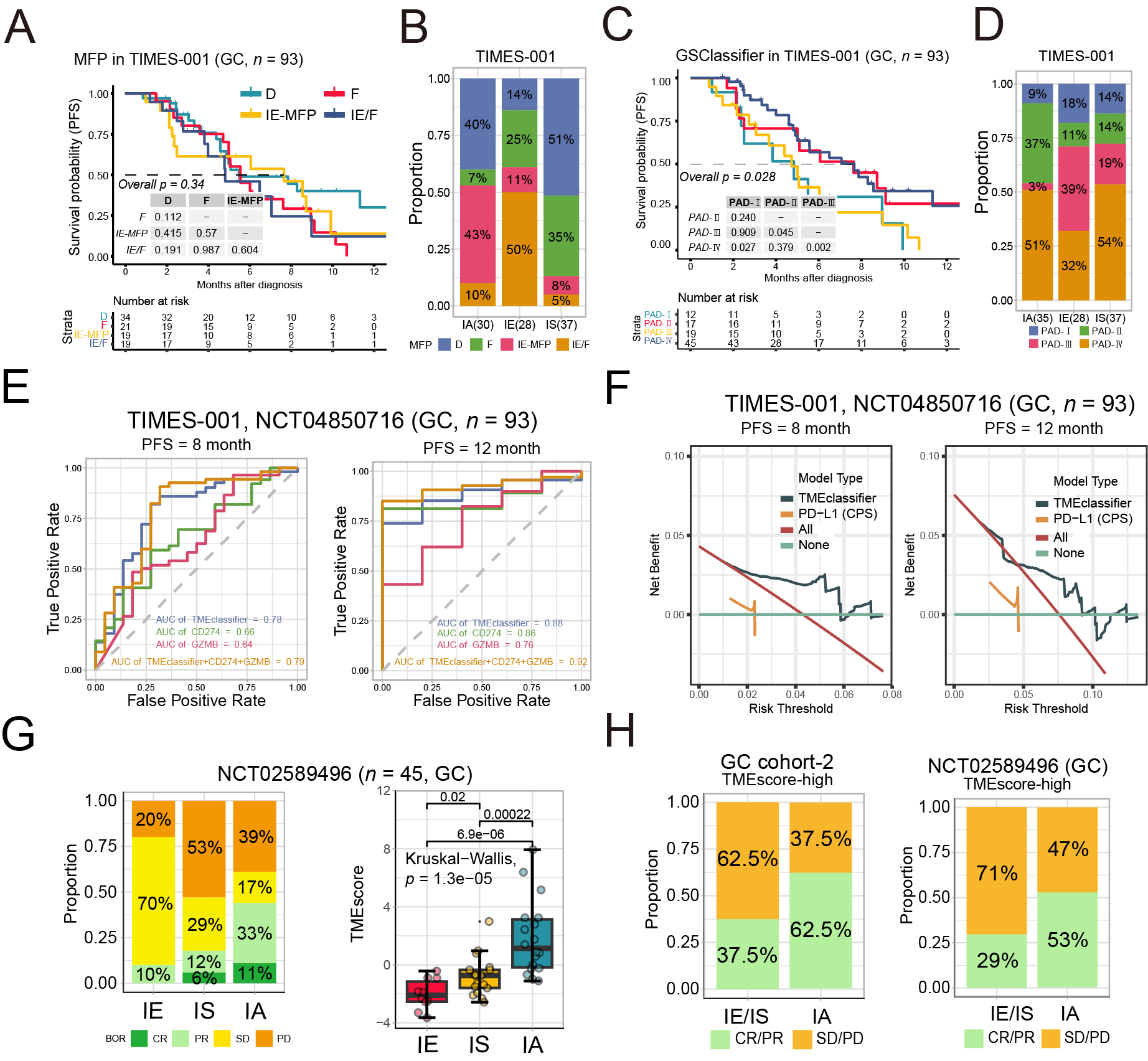
**

**Figure S5**

**A)** Kaplan–Meier curves of PFS by TME subtypes based on MFP platform of Bagave *et al.* in our TIMES001 clinical trial (*n =* 93).

**B)** Bar plots showing the relationship between the TME subtypes based on MFP platform of Bagave *et al.* and TME subtypes by TMEclassifier in our TIMES001 clinical trial.

**C)** Kaplan–Meier curves of PFS by TME subtypes based on GSCclassifier in our TIMES001 clinical trial.

**D)** Bar plots showing the relationship between the TME subtypes based on GSClassifier and TME subtypes by TMEclassifier in our TIMES001 clinical trial.

**E)** Receiver operating characteristic (ROC) analyses showing the comparison of TMEclassifier, *CD274* expression, *GZMB* expression and their combination for predicting 8 months or more PFS (left), and 12 months or more PFS (right) in the TIMES001 clinical trial.

**F)** Decision curve analysis showing the comparison of IA, PD-L1 and their combination for predicting 8 months and 12 months in PFS in the TIMES001 clinical trial.

**G)** Bar plot of immunotherapy response proportion of patients in the three TME subtypes on the left, and box plot of TMEscore of each TME subtype in Kim et al. cohort (NCT02589496, *n =* 45) on the right.

**H)** Bar plots showing the immunotherapy response proportion of patients with high TMEscore between TME subtypes in GC NanoString cohort (left) and Kim et al. cohort (right).

In bar plots, the Chi-squared test *p* values are displayed at the top of the figure. The log-rank test was used to assess the statistical significance of the prognostic differences among the subtypes in the survival analyses above. In all box plots, *p* values were calculated using the Mann–Whitney test for comparison between two groups, and the Kruskal-Wallis test was used to calculate *p* values for comparisons of more than two groups.

MFP, Molecular Functional Portrait; IE/F, immune-enriched, fibrotic; IE-MFP, immune-enriched by MFP platform;

F, fibrotic; D, immune-depleted.

**
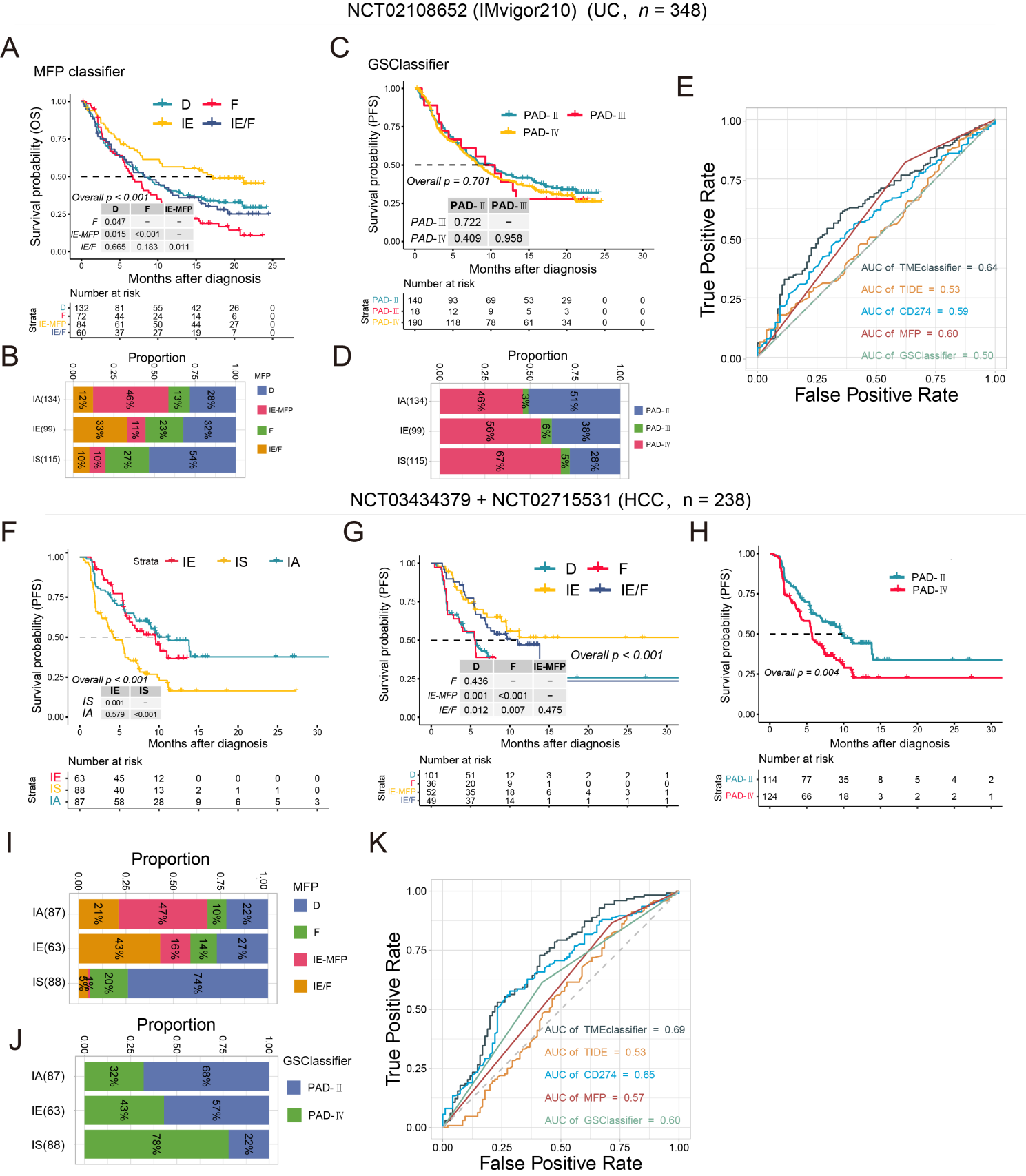
**

**Figure S6**

**A)** Kaplan–Meier curves of OS by TME subtypes by MFP in the IMvigor210 cohort (NCT02108652, *n =* 348).

**B)** Bar plots showing the relationship between the TME subtypes by MFP and TME subtypes by TMEclassifier in the IMvigor210 cohort.

**C)** Kaplan–Meier curves of PFS by TME subtypes by GSCclassifier in the IMvigor210 cohort (NCT02108652, *n =* 348).

**D)** Bar plots showing the relationship between the TME subtypes by GSCclassifier and TME subtypes by TMEclassifier in the IMvigor210 cohort.

**E)** Receiver operating characteristic analyses showing the comparison of TMEclassifier, MFP classifier, GSClassifier, TIDE, and PD-L1 gene expression for predicting PFS in the IMvigor210 clinical trial.

**F)** Kaplan–Meier curves of OS by TME subtypes of patients receiving immunotherapy from the IMbrave150 and GO30140 combined cohort (NCT03434379 and NCT02715531, *n =* 238).

**G)** Kaplan–Meier curves of PFS by TME subtypes by MFP in IMbrave150 and GO30140 cohort (NCT03434379 and NCT02715531, *n =* 238)

**H)** Kaplan–Meier curves of PFS by TME subtypes by GSClassifier in IMbrave150 and GO30140 cohort (NCT03434379 and NCT02715531, *n =* 238).

**I)** Bar plots showing the relationship between the TME subtypes by MFP and TME subtypes by TMEclassifier in the IMbrave150 and GO30140 combined cohort.

**J)** Bar plots showing the relationship between the TME subtypes by MFP and TME subtypes by TMEclassifier in the IMbrave150 and GO30140 combined cohort.

**K)** Receiver operating characteristic analyses showing the comparison of TMEclassifier, MFP classifier, GSClassifier, TIDE, and PD-L1 gene expression for predicting PFS in the IMbrave150 and GO30140 cohort.

In bar plots, the Chi-squared test *p* values are displayed at the top of the figure. The log-rank test was used to assess the statistical significance of the prognostic differences among the subtypes in the survival analyses above.

MFP, Molecular Functional Portrait; IE/F, immune-enriched, fibrotic; IE-MFP, immune-enriched by MFP platform;

F, fibrotic; D, immune-depleted.


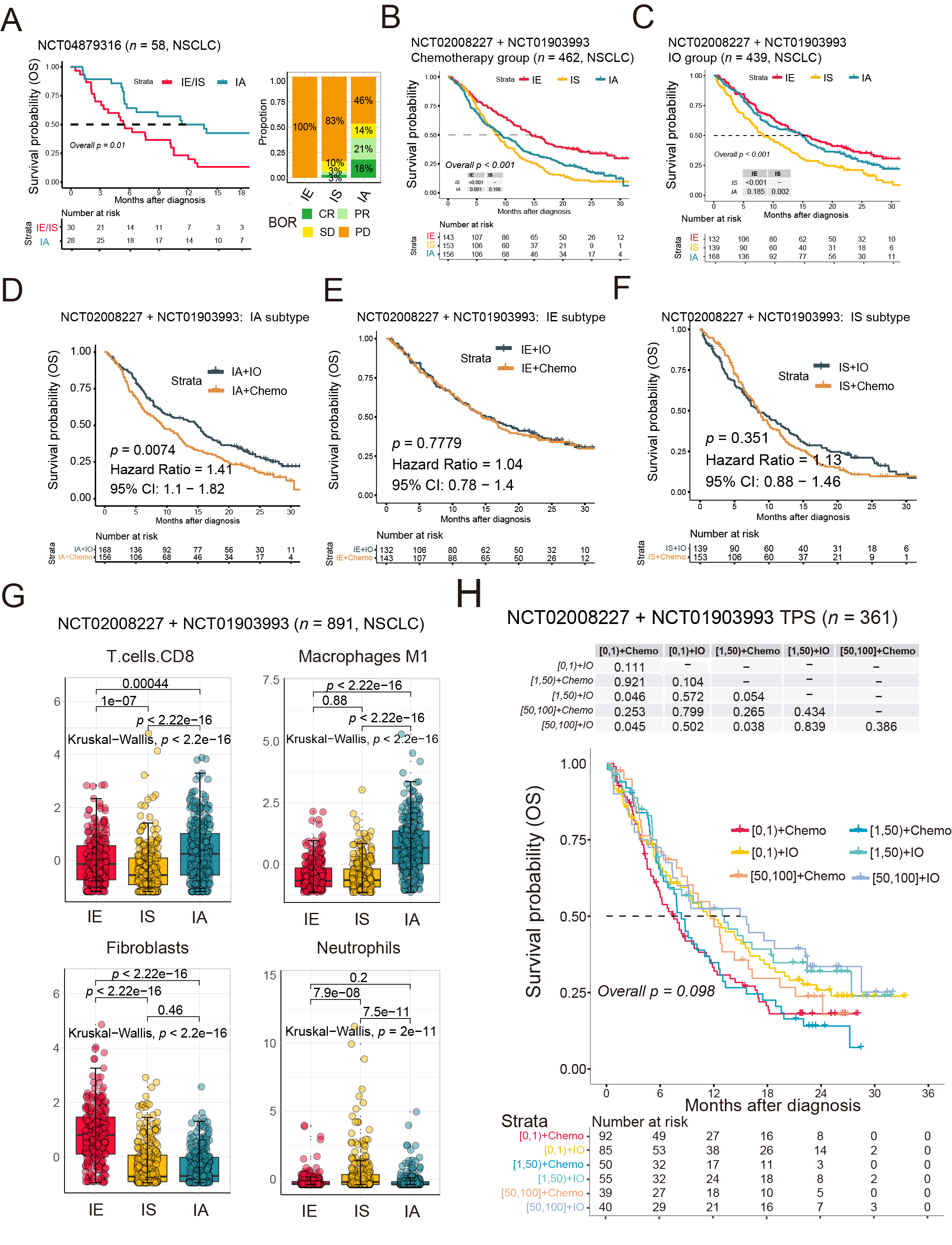


**Figure S7**

**A)** Kaplan–Meier curves of OS by TME subtypes (left), and bar plot of Immunotherapy response proportion among three TME subtypes (right) in the CERTIM cohort (NCT04879316, *n =* 58).

**B)** Kaplan–Meier curves of OS by TME subtypes in OAK and POPLAR combined cohort (NCT02008227 and NCT01903993) with chemotherapy treatment (*n* = 462).

**C)** Kaplan–Meier curves of OS by TME subtypes in OAK and POPLAR combined cohort with immunotherapy treatment (*n* = 439).

**D)** Kaplan–Meier curves of OS by different treatment regimens in IA subtype patients of OAK and POPLAR combined cohort (*n* = 324).

**E)** Kaplan–Meier curves of OS by different treatment regimens in IE subtype patients of OAK and POPLAR combined cohort (*n* = 275).

**F)** Kaplan–Meier curves of OS by different treatment regimens in IS subtype patients of OAK and POPLAR combined cohort (*n* = 292).

**G)** Comparison of four cell types among three TME subtypes in OAK and POPLAR combined cohort (*n =* 891).

**H)** Kaplan–Meier curves of OS by PD-L1 TPS with different treatments of patients from OAK and POPLAR combined cohort with TPS (*n* = 361). Each group is color-coded as: PD-L1 TPS < 1% with chemotherapy, red; PD-L1 TPS 1%~49% with chemotherapy, blue; PD-L1 TPS > 49% with chemotherapy, orange; PD-L1 TPS < 1% with immunotherapy, yellow; PD-L1 TPS 1%~49% with immunotherapy, green and PD-L1 TPS > 49% with immunotherapy, purple.

In all box plots, *p* values are calculated using Mann–Whitney test for comparison between two groups, and the Kruskal-Wallis test is used to calculate *p* values for comparisons of more than two groups. The log-rank test is used to assess the statistical significance of the prognostic differences among the subtypes in the survival analyses above.

tTMB, tissue Tumor Mutation Burden; TPS, Tumor Proportion Score.


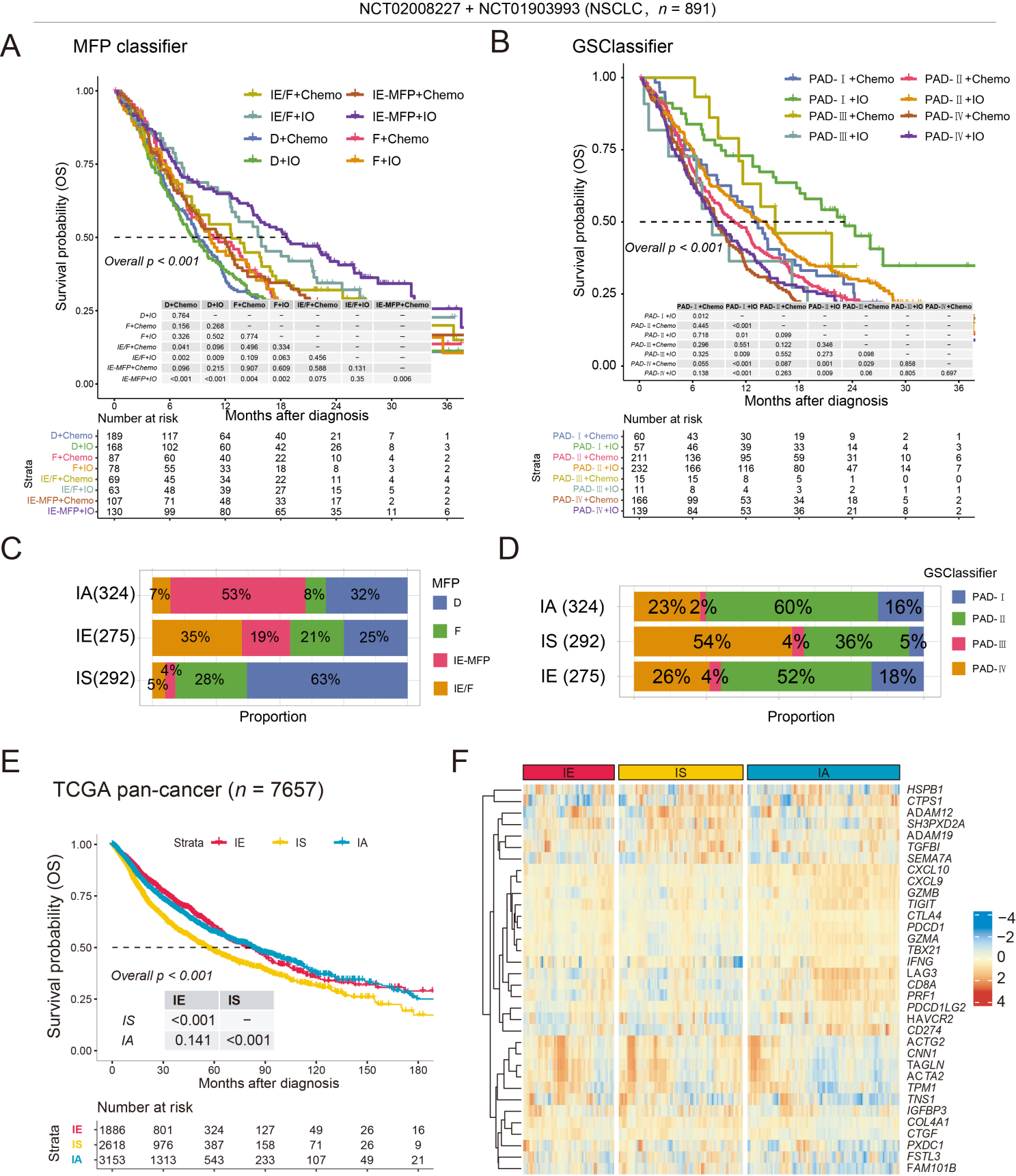


**Figure S8**

**A)** Kaplan–Meier curves of OS by TME subtypes by MFP in the OAK and POPLAR combined cohort (NCT02008227 and NCT01903993, *n =* 891).

**B)** Kaplan–Meier curves of OS by TME subtypes by GSClassifier in the OAK and POPLAR combined cohort.

**C)** Bar plots showing the relationship between the TME subtypes by MFP and TME subtypes by TMEclassifier in the OAK and POPLAR combined cohort.

**D)** Bar plots showing the relationship between the TME subtypes by GSClassifier and TME subtypes by TMEclassifier in the OAK and POPLAR combined cohort.

**E)** Kaplan–Meier curves of OS by TME subtypes of TCGA pan-cancer patients (*n =* 7657).

**F)** Heatmap showing expression of immune and stromal related genes among three TME subtypes in TCGA pan-cancer cohort.

The log-rank test assessed the statistical significance of the prognostic differences among the subtypes in the survival analyses above.
